# Novel 4,6-Disubstituted *s*-Triazin-2-yl Amino Acid Derivatives as Promising Antifungal Agents

**DOI:** 10.3390/jof6040237

**Published:** 2020-10-21

**Authors:** Rakia Abd Alhameed, Zainab Almarhoon, Essam N. Sholkamy, Salman Ali Khan, Zaheer Ul-Haq, Anamika Sharma, Beatriz G. de la Torre, Fernando Albericio, Ayman El-Faham

**Affiliations:** 1 Department of Chemistry, College of Science, King Saud University, P.O. Box 2455, Riyadh 11451, Saudi Arabia; Roki.ahmed@yahoo.com (R.A.A.); zalmarhoon@ksu.edu.sa (Z.A.); 2 Department of Botany and Microbiology, College of Science, King Saud University, P.O. Box 2455, Riyadh 11451, Saudi Arabia; essam_92003@yahoo.com; 3 Dr. Panjwani Center for Molecular medicine and Drug Research, International Center for Chemical and Biological Sciences, University of Karachi, Karachi 75270, Pakistan; salmanali9329176@gmail.com (S.A.K.); zaheer_qasmi@hotmail.com (Z.U.-H.); 4 KwaZulu-Natal Research Innovation and Sequencing Platform (KRISP), School of Laboratory Medicine and Medical Sciences, College of Health Sciences, University of KwaZulu-Natal, Durban 4041, South Africa; anamika.aug14@gmail.com (A.S.); garciadelatorreb@ukzn.ac.za (B.G.d.l.T.); 5 Peptide Science Laboratory, School of Chemistry and Physics, University of KwaZulu-Natal, Durban 4001, South Africa; 6 CIBER-BBN (Networking Centre on Bioengineering, Biomaterials and Nanomedicine) and Department of Organic Chemistry, University of Barcelona, 08028 Barcelona, Spain; 7 Institute for Advanced Chemistry of Catalonia (IQAC-CSIC), 08034 Barcelona, Spain; 8 Chemistry Department, Faculty of Science, Alexandria University, P.O. Box 426, Ibrahimia 12321, Alexandria, Egypt

**Keywords:** 2,4,6-trichlorotriazine, TCT, triazine amino acid, antifungal, *Candida albicans*, *N*-myristoltransferase

## Abstract

A novel series of 4,6-disubstituted *s*-triazin-2-yl amino acid derivatives was prepared and characterized. Most of them showed antifungal activity against *Candida albicans* compared to clotrimazole (standard drug). Compounds bearing aniline derivatives, piperidine and glycine on the triazine core showed the highest inhibition zones at concentrations of 50, 100, 200, and 300 μg per disc. In addition, docking studies revealed that all the compounds accommodated well in the active site residues of *N*-myristoltransferase (NMT) and exhibited complementarity, which explains the observed antifungal activity. Interestingly, none of these compounds showed antibacterial activity.

## 1. Introduction

An important component of a drug design program’s quest for new leads is the synthesis of molecules that are novel but mimic recognized biologically active molecules by virtue of the existence of certain crucial structural characteristics, especially in the development of several specific synthetic protocols for the preparation of a range of substituted *s*-triazine derivatives owing to their involvement in several applications in the medicinal field and the synthesis of numerous pharmaceutical agents [1,2]. The reported literature reveals that substituted *s*-triazine derivatives are consistent with a number of marked antimicrobial activities against *Staphylococcus aureus*, *Bacillus cereus*, *Bacillus subtilis*, *Salmonella typhimurium*, *Escherichia coli Klebsiella aerogenes*, and *Candida albicans* as human microbial pathogens [3].

*C. albicans* is a constituent of the normal part of the human commensal flora that lives in the human mouth and gastrointestinal tract. However, it is also is a diploid fungus that produces as both yeast and filamentous cells [4]. Systemic fungal infections (fungemias) including those by *C. albicans* have emerged as important causes of morbidity and mortality in immunocompromised patients (such as those with AIDS or under antitumor chemotherapy) [5,6]. In addition, hospital-acquired infections by *C. albicans* have become a cause of major health concerns [7,8,9,10].

In medicinal chemistry, the *s*-triazine ring has proved to be a privileged structure, and, therefore, its derivatives have been extensively studied against a broad number of biological targets. In this respect, *s*-triazine derivatives have shown antiprotozoal [11], anti-HIV [12], anticancer [13,14], antimalarial [15,16], antibacterial [17,18,19], antifungal [20,21,22], and antileishmanial activity [23], carbonic anhydrase inhibitors [24,25], and human monoamine oxidase (MAO) inhibitors [26].

Cyanuric chloride (TCT) is the starting reagent for the synthesis of *s-*triazine derivatives. TCT is a symmetrical tridentate compound (it has three reactive Cl) with the particularity from a synthetic point of view that once the first Cl has reacted, the two remaining Cl show different reactivity. This allows TCT to undergo sequential nucleophilic substitution by different nucleophiles (S, O, N) under a controlled temperature to furnish a full array of *s*-triazine derivatives [1,27,28].

Based on the previously reported data, here, we focused on the preparation of a novel series of disubstituted *s*-triazin-2-yl amino acid derivatives (**5a–q**) containing cyclic amines (morpholine, piperidine, or pyrrolidine), and 4-substituted aniline derivatives which are biologically interesting moieties [29]. To this end, we used different techniques, including conventional heating, microwave irradiation and ultrasound. The resulted disubstituted *s*-triazin-2-yl amino acid derivatives were screened against bacteria and fungi as a preliminary biological screening. In addition, molecular docking studies with *N*-Myristoltransferase (NMT) as an anti-fungal target will be discussed.

## 2. Materials and Methods

All reagents, chemicals and solvents were purchased from commercial suppliers. Reactions were monitored by using thin-layer chromatography (TLC, silica gel 60-F254 protected aluminum sheets). Melting points were conducted in open capillary tubes using a Gallenkamp melting point apparatus (Sigma-Aldrich Chemie GmbH, Taufkirchen, Germany) and were uncorrected. Fourier transform infrared spectroscopy (FTIR) spectra were recorded on a Nicolet 6700 spectrometer (Thermo Electron Scientific Instruments Corporation, Madison, WI, USA) using KBr discs. ^1^H- and ^13^C-NMR spectra were recorded on a Varian-Agilent-NMR 600 MHz spectrometer (Varian, Inc., Palo Altro, CA, USA). Elemental analyses were recorded on a Perkin-Elmer 2400 elemental analyzer (Perkin-Elmer Inc. Waltham, MA, USA). The ultrasonic bath was purchased from Selecta (Barcelona, Spain). Microwave experiments were performed in a multimode reactor (Monowave 300, Aton Paar GmbH, 1400 W maximum magnetron, Germany). Heating time (5 min at 70 °C and 350 watt) and reaction time were held for 12–15 min at identical watt and temperature with stirring speed (800 rpm); then, cooling was performed until 25 °C.

### 2.1. General Procedure for the Synthesis of Disubstituted Amino Acids-Triazine Derivatives

#### 2.1.1. One-Pot Synthesis

A mixture of amino acid (10 mmol) and NaHCO_3_ (22 mmol) in 50 mL distilled water was added dropwise to a solution of cyanuric chloride (TCT; **1**, 10 mmol) in 50 mL acetone at 0 °C. The reaction mixture was kept under stirring at 0 °C for 2 h, and then a solution of the amine (10 mmol) in 10 mL acetone was added dropwise (5 min) at 0 °C, followed by addition of a solution of NaHCO_3_ (11 mmol) in 20 mL distilled water. After complete addition, the reaction mixture was stirred overnight at room temperature. The solvent was concentrated under vacuum and then extracted with dichloromethane (10 mL). The aqueous layer was collected and acidified with 1N HCl. The white precipitate of the products (**4a–f**) was collected by filtration.

#### 2.1.2. Stepwise Synthesis

The method was performed in the following two steps: Step (i) synthesis of monosubstituted *s*-triazine **3**: a solution of the amine (1 equiv.) in 10 mL acetone was added dropwise at 0 °C in 5 min to a mixture of cyanuric chloride (1 equiv.) in 50 mL acetone, followed by the addition of a solution of NaHCO_3_ (1.5 Equation.) in 20 mL distilled water. The reaction mixture was kept under stirring at 0 °C for 2 h to afford mono substituted *s*-triazine derivatives. Step (ii) synthesis of disubstituted amino acid s-triazine: a mixture of amino acid (1 equiv.) and NaHCO_3_ (2.5 eq.) in 50 mL distilled water was added dropwise (10 min) to a solution of the previously prepared monosubstituted *s*-triazine **3** (1 equiv.) in 50 mL acetone; the reaction mixture was stirred at room temperature overnight and then acidified with 1N HCl to furnish the target compounds **4a–f**.

### 2.2. General Procedures for the Synthesis of 4,6,-Disubstituted s-Triazin-2-yl Amino Acid Derivatives ***5a–q***

**Method A**: Conventional heating

Disubstituted *s*-triazine (1 mmol) was reacted with different amines (morpholine, piperidine and pyrrolidine; 1.1 mmol) in tetrahydrofuran (THF, 20 mL) and in the presence of diisopropylethylamine (DIEA; 2.2 mmol) under reflux for 24 h. The solvent was evaporated under vacuum to produce a white precipitate, which was neutralized by 1N HCl to afford trisubstituted *s-*triazine derivatives in 89–93% yield.

**Method B**: Microwave technique

Disubstituted *s*-triazine (1 mmol) was dissolved in 5 mL THF, and then 1.1 mmol of amine was added, followed by DIEA (2.2 mmol). This mixture was then subjected to microwave irradiation using a multimode reactor (Monowave 300, Aton Paar GmbH), where heating time (5 min, at 70 °C, 350 watt) and the reaction were held for 12–15 min at the same wattage and temperature with a stirring speed of 800 rpm. After cooling, the solvent was evaporated, and the resulting residue was subjected to neutralization with 1N HCl to obtain trisubstituted *s*-triazine derivatives in excellent yields and purities.

**Method C**: Ultrasonication method

An amine (1.1 mmol) was added to a solution of disubstituted-triazine (1 mmol) in 20 mL THF in the presence of DIEA (2.2 mmol), and the reaction mixture was subjected to ultrasound irradiation (1.5 h) at room temperature. TLC was used to monitor the completion of reaction, followed by workup similar to conventional methodology to obtain the product with highly yield (90–93%) and purity.

All compounds obtained from the three methods were purified via washing with a hot mixture of THF and a few drops of acetone.

#### 2.2.1. (4-Morpholino-6-(Phenylamino)-1,3,5-Triazin-2-yl) Glycine (5a)

White solid in 89% (Method A), 91% (Method B) 92% (Method C) yield; mp 245–247 °C. IR (KBr, cm^−1^): 3341 (NH); 1669 (CO); ^1^H-NMR (DMSO-d_6_; ppm): δ 3.60 (4H, s, CH_2_-O-CH_2_), 3.66 (4H, s, CH_2_-N-CH_2_), 3.87 (2H, dd, *J* = 5.4 Hz, CH_2_COOH), 6.90 (1H, s.br, H_4`_), 7.19–7.23 (3H, m, H_3`_, H_5`_& NH), 7.68 (2H, dd, *J* = 7.2 Hz, H_2`_ & H_6`_), 9.08 (1H, s, NH); ^13^C-NMR (DMSO-d_6_; ppm): δ 42.5 (CH_2_COOH); 43.4 (CH_2_-N-CH_2_), 66.2 (CH_2_-O-CH_2_), 119.7 (C_2`_ & C_6`_); 121.6 (C_4`_); 128.4 (C_3`_& C_5`_); 140.4 (C_1`_); 164.2, 164.8 & 165.9 (triazine), 172.3 (CO). Anal. Calcd for C_15_H_18_N_6_O_3_ (330.35): C, 54.54; H, 5.49; N, 25.44. Found: C, 54.22; H, 5.56; N, 25.63; HRMS: *m*/*z*: calcd. 331.35[M+H]^+^; Found: 331.08.

#### 2.2.2. (4-((4-Chlorophenyl) Amino)-6-Morpholino-1,3,5-Triazin-2-yl) Glycine (5b)

White powder in 87% (Method A), 90% (Method B), 92% (Method C) yield; mp 246–248 °C. IR (KBr, cm^−1^): 3350 & 3412 (NH); 1678 (CO). ^1^H-NMR (DMSO-d_6_; ppm): δ 3.60 (4H, s, CH_2_-O-CH_2_), 3.65 (4H, s, CH_2_-N-CH_2_), 3.87 (2H, dd, *J* = 4.8, 5.4 Hz, CH_2_COOH), 7.23–7.27 (3H, m, H_3`_, H_5`_& NH), 7.71 (2H, dd, *J* = 7.2, 8.4 Hz, H_2`_ & H_6`_), 9.25 (1H, s, NH); ^13^C-NMR (DMSO-d_6_; ppm): δ 42.7 (CH_2_COOH); 43.7 (CH_2_-N-CH_2_), 66.4 (CH_2_-O-CH_2_), 121.5 (C_2`_ & C_6`_); 125.4 (C_4`_); 128.5 (C_3`_& C_5`_); 139.7 (C_1`_); 164.2, 164.9 & 165.9 (triazine), 172.5 (CO). Anal. Calcd for C_15_H_17_ClN_6_O_3_ (364.79): C, 49.39; H, 4.70; N, 23.04. Found: C, 49.54; H, 4.81; N, 23.19; HRMS: *m*/*z*: calcd. 365.80[M+H]^+^, Found: 365.64.

#### 2.2.3. (4-((4-Methoxyphenyl) Amino)-6-Morpholino-1,3,5-Triazin-2-yl) Glycine (5c)

Off-white powder in 86% (Method A), 90% (Method B), 90% (Method C) yield; mp 246–248 °C. IR (KBr, cm^−1^): 3349 (NH); 1674 (CO);^1^H-NMR (DMSO-d_6_; ppm): δ 3.59 (4H, s, CH_2_-O-CH_2_), 3.64 (4H, s, CH_2_-N-CH_2_), 3.69 (3H, s, OCH_3_), 3.83 (2H, d, *J* = 5.4 Hz, CH_2_COOH), 6.80 (2H, t, *J* = 7.8, 10.8 Hz, H_3`_& H_5`_), 7.54 (2H, d, *J* = 9 Hz, H_2`_ & H_6`_), 7.57 (1H, s, NH), 8.91 (1H, s, NH); ^13^C-NMR (DMSO-d_6_; ppm): δ 42.7 (CH_2_COOH); 43.6 (CH_2_-N-CH_2_), 55.4 (OCH_3_), 66.3 (CH_2_-O-CH_2_), 113.8 (C_3`_ & C_5`_); 121.6 (C_2`_& C_6`_); 133.7 (C_1`_); 154.5 (C_4`_); 164.1, 165.0 & 166.0 (triazine), 172.6 (CO). Anal. Calcd for C_16_H_20_N_6_O_4_ (360.37): C, 53.33; H, 5.59; N, 23.32. Found: C, 53.54; H, 5.71; N, 23.17; HRMS: *m*/*z*: calcd. 361.38 [M+H]^+^; Found: 361.13.

#### 2.2.4. (4-(Phenylamino)-6-(Piperidin-1-yl)-1,3,5-Triazin-2-yl) Glycine (5d)

White powder in 89% (Method A), 93% (Method B), 92% (Method C) yield; mp 268–270 °C. IR (KBr, cm^−1^): 3339 (NH); 1674 (CO);^1^H-NMR (DMSO-d_6_; ppm): δ1.46 (4H, s, H_3`_ & H_5`_), 1.59 (2H, s, H_4`_),3.67 (4H, s, CH_2_-N-CH_2_ ), 3.86 (2H, dd, *J* = 5.4, 6 Hz, NHCH_2_COOH), 6.88 (1H, t, *J* = 7.2, 7.8 Hz, H_4``_), 7.08 (1H, s, NH), 7.20 (2H, q, *J* = 7.8, 8.4 Hz, H_3``_ & H_5``_), 7.69 (2H, dd, *J* = 7.2, 8.4 Hz, H_2``_ & H_6``_), 8.96 (1H, d, NH); ^13^C-NMR (DMSO-d_6_; ppm): δ24.5 (C_4`_), 25.6(C_3`_& C_5`_), 42.5 (HOOC-CH_2_NH), 43.7 (C_2`_& C_6`_); 119.6 (C_2``_ & C_6``_); 121.4 (C_4``_); 128.4 (C_3``_& C_5``_); 140.6 (C_1``_); 164.1, 165. 8, 166.2 (triazine), 172.4 (CO). Anal. Calcd for C_16_H_20_N_6_O_2_ (328.38): C, 58.52; H, 6.14; N, 25.59. Found: C, 58.41; H, 6.10; N, 25.71. HRMS: *m*/*z*: calcd. 329.38 [M+H]^+^, Found: 329.13.

#### 2.2.5. (4-((4-Chlorophenyl) Amino)-6-(Piperidin-1-yl)-1,3,5-Triazin-2-yl) Glycine (5e)

White powder in 88% (Method A), 91% (Method B), 92% (Method C) yield; mp 260–262 °C. IR (KBr, cm^−1^): 3396 (NH); 1654 (CO); ^1^H-NMR (DMSO-d_6_; ppm): δ 1.46 (4H, s, H_3`_& H_5`_), 1.59 (2H, s, H_4`_), 3. 67 (4H, s, CH_2_-N-CH_2_), 3.85 (2H, dd, *J* = 4.8 Hz, CH_2_COOH), 7.15 (1H, s, NH), 7.24 (2H, t, *J* = 9,12.6 Hz, H_3``_ & _H5``_), 7.73 (2H, dd, *J* = 7.2, 7.8Hz, H_2``_ & H_6``_), 9.13 (1H, s, NH); ^13^C-NMR (DMSO-d_6_; ppm): δ24.8 (C_4`_), 25.8 (C_3`_& C_5`_), 42.8 (HOOC-CH_2_NH), 44.0 (C_2`_& C_6`_); 121.3 (C_2``_ & C_6``_); 125.1 (C_4``_); 128.5 (C_3``_& C_5``_); 139.9 (C_1``_); 164. 3, 166.1, 166.5 (triazine), 172.6 (CO).Anal. Calcd for C_16_H_19_ClN_6_O_2_ (362.82): C, 52.97; H, 5.28; N, 23.16. Found: C, 53.09; H, 5.32; N, 23.40. HRMS: *m*/*z*: calcd. 363.83 [M+H]^+^; Found: 363.80.

#### 2.2.6. (4-((4-Methoxyphenyl) Amino)-6-(Piperidin-1-yl)-1,3,5-Triazin-2-yl) Glycine (5f)

Off-white powder in 87% (Method A), 90% (Method B), 90% (Method C) yield; mp 261–263 °C. IR (KBr, cm^−1^): 3260 (NH); 1667 (CO); ^1^H-NMR (DMSO-d_6_; ppm): δ 1.45 (4H, s, H_3`_& H_5`_), 1.59 (2H, s, H_4`_), 3. 66 (4H, s, CH_2_-N-CH_2_), 3.68 (3H, s, OCH_3_), 3.84 (2H, dd, CH_2_COOH), 6.80 (2H, dd, *J* = 9, 10.8 Hz, H_3``_ & H_5``_), 7.01 (1H, s, NH), 7.57 (2H, dd, *J* = 6, 8.4Hz, H_2``_ & H_6``_), 8.81 (1H, d, NH);^13^C-NMR (DMSO-d_6_; ppm): δ 24.8 (C_4`_), 25.8 (C_3`_& C_5`_), 42.8 (CH_2_COOH), 44.0 (C_2`_& C_6`_); 55.6 (OCH_3_), 113.9 (C_3``_& C_5``_), 121.39 (C_2``_ & C_6``_); 134.0 (C_1``_); 154.4 (C_4``_), 164. 3, 164.5, 166.4 (triazine), 172.7 (CO). Anal. Calcd for C_17_H_22_N_6_O_3_ (358.40): C, 56.97; H, 6.19; N, 23.45. Found C, 56.72; H, 6.30; N, 23.67. HRMS: *m*/*z*: calcd. 359.41 [M+H]^+^; Found: 359.10.

#### 2.2.7. (4-((4-Chlorophenyl) Amino)-6-(Pyrrolidin-1-yl)-1,3,5-Triazin-2-yl) Glycine (5g)

White powder in 86% (Method A), 92% (Method B), 91% (Method C) yield; mp 262–264 °C. IR (KBr, cm^−1^): 3371, 3246 (NH); 1661 (CO). ^1^H-NMR (DMSO-d_6_; ppm): δ 1.86 (4H, s, H_3``_ & H_4``_), 3.39–3.46 (4H, m, H_2``_ & H_5``_), 3.86 (2H, d, *J* = 5.4 Hz, NH CH_2_COOH), 7.10 (1H, d, NH). 7.23 (2H, t, *J* = 9, 9.6 Hz, H_3`_ & H_5`_), 7.80 (2H, dd, *J* = 7.2, 8.4 Hz, H_2`_ & H_6`_), 9.13 (1H, d, NH);^13^C-NMR (DMSO-d_6_; ppm): δ 25.3 (C_3``_ & C_4``_), 42.7 (NHCH_2_COOH), 46.5 (C_2``_ & C_5``_), 121.6 (C_2`_ & C_6`_), 125.8 (C_4`_), 128.6 (C_3`_& C_5`_), 139.8 (C_1`_); 161.3, 163.1, 164.7 (triazine), 172.5 (CO).Anal. Calcd for C_15_H_17_ClN_6_O_2_ (348.79): C, 51.65; H, 4.91; N, 24.10. Found C, 51.81; H, 5.02; N, 24.01. HRMS: *m*/*z*: calcd. 349.80 [M+H]^+^; Found: 349.63.

#### 2.2.8. (4-((4-Methoxyphenyl) Amino)-6-(Pyrrolidin-1-yl)-1,3,5-Triazin-2-yl) Glycine (5h)

White powder in 86% (Method A), 90% (Method B), 93% (Method C) yield; mp 270–272 °C. IR (KBr, cm^−1^): 3262 (NH); 1668 (CO);^1^H-NMR (DMSO-d_6_; ppm): δ 1.85 (4H, s, H_3``_ & H_4``_), 3.44 (4H, s, H_2``_ & H_5``_), 3.68 (3H, s, OMe), 3.86 (2H, s, NH-CH_2_COOH), 6.79 (2H, d, *J* = 7.8 Hz, H_3`_ & H_5`_), 6.90 (1H, s, NH), 7.66 (2H, d, *J* = 7.8 Hz*,* H_2`_ & H_6`_), 8.78 (1H, d, NH); ^13^C-NMR (DMSO-d_6_; ppm): δ 25.3 (C_3``_& C_4``_), 42.7 (CH_2_COOH), 46.1 (C_2``_ & C_5``_), 55.6 (OMe), 113.9 (C_3`_ & C_5`_), 121.2 (C_2`_& C_6`_), 134.4 (C_1`_); 154.3 (C_4`_), 163.5, 165.8, 166.1 (triazine), 172.7 (CO). Anal. Calcd for C_16_H_20_N_6_O_3_ (344.38): C, 55.80; H, 5.85; N, 24.40. Found: C, 55.99; H, 5.97; N, 24.64. HRMS: *m*/*z*: calcd. 345.38 [M+H]^+^; Found: 345.56.

#### 2.2.9. 3-((4-Morpholino-6-(Phenylamino)-1,3,5-Triazin-2-yl) Amino) Propanoic Acid (5i)

White powder in 84% (Method A), 91% (Method B), 93% (Method C) yield; mp 267–269 °C. IR (KBr, cm^−1^): 3285 (NH); 1666 (CO). ^1^H-NMR (DMSO-d_6_; ppm): δ 2.50 (2H, t, *J* = 7.2 Hz, CH_2_COOH), 3.45 (2H, q, *J* = 10.8 HZ, NH-CH_2_CH_2_), 3.60 (4H, dd, *J* = 7.2 Hz, CH_2_-O-CH_2_), 3.66 (4H, dd, CH_2_-N-CH_2_), 6.89 (1H, s, H_4`_), 6.93 (1H, s, NH). 7.21 (2H, d, *J* = 7.8 Hz, H_3`_ & H_5`_), 7.71(2H, d, *J* = 7.8 Hz, H_2`_ & H_6`_), 9.00 (1H, d, NH), 12.19 (1H, s, COOH);^13^C-NMR (DMSO-d_6_; ppm): δ 34.5 (CH_2_CH_2_COOH); 36.8 (NH CH_2_CH_2_COOH), 43.7 (CH_2_-N-CH_2_), 66.5 (CH_2_-O-CH_2_), 119.9 (C_2`_ & C_6`_); 121.7 (C_4`_); 128.7 (C_3`_& C_5`_); 140.9 (C_1`_); 164.4, 165.1 & 165.9 (triazine), 173.6 (CO). Anal. Calcd for C_16_H_20_N_6_O_3_ (344.38): C, 55.80; H, 5.85; N, 24.40. Found: C, 55.80; H, 5.85; N, 24.40. HRMS: *m*/*z*: calcd. 345.38 [M+H]^+^; Found: 345.44.

#### 2.2.10. 3-((4-((4-Chlorophenyl) Amino)-6-Morpholino-1,3,5-Triazin-2-yl) Amino) Propanoic Acid (5j)

White powder in 86% (Method A), 93% (Method B), 90% (Method C) yield; mp 285–287 °C. IR (KBr, cm^−1^): 3277 (NH); 1678 (CO). ^1^H-NMR (DMSO-d_6_; ppm): δ2.48 (2H, s, CH_2_COOH), 3.44 (2H, q, *J* = 7.2 Hz, NH-CH_2_CH_2_), 3.60 (4H, d, *J* = 7.2 Hz, CH_2_-O-CH_2_), 3.64 (4H, dd, CH_2_-N-CH_2_), 6.99 (1H, s, NH), 7.24 (2H, d, *J* = 7.8 Hz, H_3`_ & H_5`_), 7.75 (2H, d, *J* = 7.2 HZ, H_2`_ & H_6`_), 9.17 (1H, d, NH); ^13^C-NMR (DMSO-d_6_; ppm): δ 34.4 (CH_2_CH_2_COOH), 36.8 (NHCH_2_CH_2_); 43.7 (CH_2_-N-CH_2_), 66.4 (CH_2_-O-CH_2_), 121.3 (C_2`_& C_6`_); 125.3 (C_4`_); 128.6 (C_3`_& C_5`_); 140.0 (C_1`_); 164.3, 165.0 & 165.8 (triazine), 173.5 (CO). Anal. Calcd for C_16_H_19_ClN_6_O_3_ (378.82): C, 50.73; H, 5.06; N, 22.19. Found: C, 50.95; H, 5.23; N, 22.45. HRMS: *m*/*z*: calcd. 379.82 [M+H]^+^; Found: 379.76.

#### 2.2.11. 3-((4-((4-Methoxyphenyl) Amino)-6-Morpholino-1,3,5-Triazin-2-yl) Amino) Propanoic Acid (5k)

Off-white powder in 88% (Method A), 90% (Method B), 92% (Method C) yield; mp 269–271 °C. IR (KBr, cm^−1^): 3273 (NH); 1667 (CO).^1^H-NMR (DMSO-d_6_; ppm): δ 2.49 (2H, s, CH_2_COOH), 3.43 (2H, d, *J* = 4.2 Hz, NHCH_2_CH_2_), 3.59–3.66 (8H, m, CH_2_-O-CH_2_ & CH_2_-N-CH_2_), 3.69 (3H, s, OCH_3_), 6.79 (1H, s, NH), 6.80 (2H, d, *J* = 8.4 Hz, H_3`_& H_5`_), 7.57 (2H, s, H_2`_ & H_6`_), 8.83 (1H, d, NH); ^13^C-NMR (DMSO-d_6_; ppm): δ 34.7 (CH_2_COOH); 37.0 (NHCH_2_CH_2_COOH), 44.0 (CH_2_-N-CH_2_), 55.7 (OCH_3_), 66.7 (CH_2_-O-CH_2_), 114.1 (C_3`_ & C_5`_); 121.9 (C_2`_& C_6`_); 134.1 (C_1`_); 154. 8 (C_4`_); 164.5, 165.3 & 165.9 (triazine), 173.89 (CO). Anal. Calcd for C_17_H_22_N_6_O_4_ (374.40): C, 54.54; H, 5.92; N, 22.45. Found: C, 54.75; H, 6.08; N, 22.69. HRMS: *m*/*z*: calcd. 375.40 [M+H]^+^; Found: 375.61.

#### 2.2.12. 3-((4-(Phenylamino)-6-(Piperidin-1-yl)-1,3,5-Triazin-2-yl) Amino) Propanoic Acid (5l)

White powder in 89% (Method A); 92% (Method B), 94% (Method C) yield; mp 276–278 °C. IR (KBr, cm^−1^): 3275 (NH); 1663 (CO).^1^H-NMR (DMSO-d_6_; ppm): δ 1.46 (4H, s, H_3`_ & H_5`_), 1.59 (2H, s, H4^`^), 2.49 2H, s, CH_2_ COOH), 3.45 (2H, t, *J* = 6, 10.8 Hz, CH_2_NH), 3.66 (4H, d, CH_2_-N-CH_2_), 6.83 (1H, s, NH), 6.87 (1H, s, H_4``_), 7.20 (2H, t, *J* = 7.2,7.8 Hz, H_3``_ & H_5``_), 7.72(2H, d, *J* = 7.8 Hz, H_2``_ & H_6``_), 8.92 (1H, d, NH);^13^C-NMR (DMSO-d_6_; ppm): δ 24.6 (C_4`_), 25.3 (C_3`_& C_5`_), 34.3 (CH_2_-COOH), 36.5 (NHCH_2_CH_2_COOH), 43.7 (C_2`_& C_6`_); 119.5 (C_2``_ & C_6``_); 121.3 (C_4``_); 128.4 (C_3``_& C_5``_); 140.8 (C_1``_); 164. 4, 165.7, 165.9 (triazine), 173.4 (CO). Anal. Calcd for C_17_H_22_N_6_O_2_ (342.40): C, 59.63; H, 6.48; N, 24.54. Found: C, 59.89; H, 6.66; N, 24.87. HRMS: *m*/*z*: calcd. 343.40 [M+H]^+^; Found: 343.61.

#### 2.2.13. 3-((4-((4-Chlorophenyl) Amino)-6-(Piperidin-1-yl)-1,3,5-Triazin-2-yl) Amino) Propanoic Acid (5m)

White powder in 86% (Method A) 92% (Method B) 92% (Method C) yield; mp 266–268 °C. IR (KBr, cm^−1^): 3262 (NH); 1665 (CO); ^1^H-NMR (DMSO-d_6_; ppm): δ 1.46 (4H, s, H_3`_ & H_5`_), 1.59 (2H, s, H_4`_), 2.50 (2H, s, CH_2_COOH), 3.43 (2H, q, *J* = 6.6 Hz, CH_2_CH_2_NH), 3.67 (4H, d, CH_2_-N-CH_2_), 6.89 (1H, d, NH), 7.24 (2H, d, *J* = 7.8 Hz, H_3``_& H_5``_), 7.76 (2H, d, *J* = 8.4 Hz, H_2``_ & H_6``_), 9.09 (1H, d, NH);^13^C-NMR (DMSO-d_6_; ppm): δ24.8 (C_4`_), 25.8 (C_3`_& C_5`_), 34.4 (CH_2_CH_2_COOH), 36.8 (CH_2_CH_2_NH), 44.0 (C_2`_& C_6`_); 121.2 (C_2``_ & C_6``_); 125.0 (C_4``_); 128.5 (C_3``_& C_5``_); 140.1 (C_1``_); 164. 5, 165.8, 166.1 (triazine), 173.5 (CO). Anal. Calcd for C_17_H_21_ClN_6_O_2_ (376.85): C, 54.18; H, 5.62; N, 22.30. Found: C, 54.33; H, 5.76; N, 22.54. HRMS: *m*/*z*: calcd. 377.85 [M+H]^+^; Found: 377.63.

#### 2.2.14. 3-((4-((4-Methoxyphenyl) Amino)-6-(Piperidin-1-yl)-1,3,5-Triazin-2-yl) Amino) Propanoic Acid (5n)

Off-white powder in 89% (Method A) 94% (Method B) 93% (Method C) yield; mp 275–277 °C. IR (KBr, cm^−1^): 3259 (NH); 1667 (CO); ^1^H-NMR (DMSO-d_6_; ppm): δ 1.45 (4H, s, H_3`_ & H_5`_), 1.58 (2H, s, H_4`_), 2.50 (2H, s, CH_2_COOH), 3. 43 (2H, s, HOOC-CH_2_CH_2_NH), 3.65 (4H, s, CH_2_-N-CH_2_), 3.68 (3H, s, OCH_3_), 6.79 (2H, d, *J* = 9 Hz, H_3``_ & H_5``_), 7.59 (2H, s, H_2``_ & H_6``_), 8.84 (1H, d, NH);^13^C-NMR (DMSO-d_6_; ppm): δ24.9 (C_4`_), 25.8 (C_3`_& C_5`_), 34.6 (CH_2_COOH), 36.8 (CH_2_CH_2_NH) 43.9 (C_2`_& C_6`_); 55.5 (OCH_3_), 113.9 (C_3``_& C_5``_), 121.2 (C_2``_ & C_6``_); 134.2 (C_1``_); 154.4 (C_4``_), 164. 4, 164.7, 166.0 (triazine), 173.6 (CO). Anal. Calcd for C_18_H_24_N_6_O_3_ (372.43): C, 58.05; H, 6.50; N, 22.57. Found: C, 58.23; H, 6.62; N, 22.81. HRMS: *m*/*z*: calcd. 373.43 [M+H]^+^; Found: 373.55.

#### 2.2.15. 3-((4-(Phenylamino)-6-(Pyrrolidin-1-yl)-1,3,5-Triazin-2-yl) Amino) Propanoic Acid (5o)

White powder in 87% (Method A) 91% (Method B) 92% (Method C) yield, mp 291–293°C. IR (KBr, cm^−1^): 3268 (NH); 1670 (CO);^1^H-NMR (DMSO-d_6_): δ 1.86 (4H, s, H_3``_ & H_4``_), 2.51 (2H, s, CH_2_COOH), 3.46 (6H, s, H_2``_, H_5``_ & NHCH_2_CH_2_COOH), 6.87 (1H, d, *J* = 6 Hz, H_4`_),7.19 (2H, d, *J* = 7.8 Hz, H_3`_ & H_5`_), 7.79 (2H, s*,* H_2`_ & H_6`_), 8.91 (1H, s, NH), 12.18 (1H, s, COOH); ^13^C-NMR (DMSO-d_6_; ppm): δ 25.3 (C_3``_& C_4``_), 34.5 (CH_2_COOH), 36.8 (NHCH_2_CH_2_COOH), 46.1 (C_2``_ & C_5``_), 119.6 (C_2`_ & C_6`_), 121.4 (C_4`_), 128.7 (C_3`_& C_5`_), 141.3 (C_1`_), 163.7, 164. 2 (triazine), 173.6 (CO). Anal. Calcd for C_16_H_20_N_6_O_2_ (328.38): C, 58.52; H, 6.14; N, 25.59. Found: C, 58.52; H, 6.14; N, 25.59. HRMS: *m*/*z*: calcd. 329.38 [M+H]^+^; Found: 329.35.

#### 2.2.16. 3-((4-((4-Chlorophenyl) Amino)-6-(Pyrrolidin-1-yl)-1,3,5-Triazin-2-yl) Amino) Propanoic Acid (5p)

White powder in 89% (Method A), 92% (Method B) 90% (Method C) yield; mp 289–291 °C. IR (KBr, cm^−1^): 3270 (NH); 1676 (CO);^1^H-NMR (DMSO-d_6_; ppm): δ 1.85 (4H, s, H_3``_ & H_4``_), 2.48 (2H, s, CH_2_COOH), 3.38 (6H, s, CH_2_CH_2_NH, H_2``_ & H_5``_), 3.45 (4H, d, *J* = 5.4 Hz*,* H_2``_ & H_5``_), 6.85 (1H, d, NH), 7.23 (2H, d, *J* = 7.2 Hz, H_3`_ & H_5`_), 7.82 (2H, d, *J* = 8.4 Hz*,* H_2`_ & H_6`_), 9.09 (1H, d, NH); ^13^C-NMR (DMSO-d_6_; ppm): δ 25.3 (C_3``_& C_4``_),34.5 (CH_2_COOH), 36.8 (CH_2_CH_2_NH), 46.1 (C_2``_ & C_5``_), 121.1 (C_2`_ & C_6`_), 124.9 (C_4`_), 128.5 (C_3`_ & C_5`_), 140.3 (C_1`_); 163.6, 164.1, 165.8 (triazine), 173.5 (CO). Anal. Calcd for C_16_H_19_ClN_6_O_2_ (362.82): C, 52.97; H, 5.28; N, 23.16. C, 52.76; H, 5.34; N, 23.43. HRMS: *m*/*z*: calcd. 363.82 [M+H]^+^; Found: 363.71.

#### 2.2.17. 3-((4-((4-Methoxyphenyl) Amino)-6-(Pyrrolidin-1-yl)-1,3,5-Triazin-2-yl) Amino) Propanoic Acid (5q)

White powder in 86% (Method A), 90% (Method B), 92% (Method C) yield; mp 273–275 °C. IR (KBr, cm^−1^): 3261 (NH); 1660 (CO); ^1^H-NMR (DMSO-d_6_; ppm): δ 1.84 (4H, s, H_3``_ & H_4``_), 2.49 (CH_2_COOH), 3.39–344 (6H, m, H_2``_, H_5``_& NHCH_2_CH_2_COOH), 3.68 (3H, s, OMe), 6.79 (2H, d, *J* = 9 Hz, H_3`_ & H_5`_), 7.66 (2H, d, *J* = 9.6 Hz*,* H_2`_ & H_6`_), 8.73 (1H, d, NH); ^13^C-NMR (DMSO-d_6_): δ 25.2 (C_3``_& C_4``_), 34.6 (CH_2_COOH), 36.8 (NHCH_2_CH_2_COOH), 46.1 (C_2``_ & C_5``_), 55.5 (OMe), 113.9 (C_3`_ & C_5`_), 121.1 (C_2`_& C_6`_), 134.4 (C_1`_), 154.3 (C_4`_); 163.7, 164. 0, 165.7 (triazine), 173.6 (CO).Anal. Calcd for C_17_H_22_N_6_O_3_ (358.40): C, 56.97; H, 6.19; N, 23.45. Found: C, 57.12; H, 6.31; N, 23.29. HRMS: *m*/*z*: calcd. 359.40 [M+H]^+^; Found: 359.53.

### 2.3. Antimicrobial Activity

#### 2.3.1. Preparation of Microbial Inoculums

The antibacterial activity of **5a–q** was evaluated against Gram-positive bacteria “*S. aureus* ATCC 29213 and *S. epidermidis* ATCC 12228”, Gram-negative bacteria “*E. coli* ATCC 25922 and *S. typhimurium* ATCC 14028” and *C. albicans* ATCC 60193.

#### 2.3.2. Antimicrobial Assay Using the Disc Diffusion Method

The microbial inoculums were prepared as described in our previous work [30]. The antibacterial activity of **5a–q** was studied by employing a micro-dilution method, using Mueller–Hinton broth and following the guidelines of the Clinical and Laboratory Standards Institute (CLSI) (M07, 11th Edition, 2018) [31], and serial dilution was performed using dimethyl sulfoxide (DMSO) to prepare 200 μg/mL of each compound. Paper discs (6 mm diameter) previously dipped into each compound were placed gently onto the microorganism-seeded plates. The antibacterial assay plates were then incubated at 24 h at 37 ± 1 °C. The diameters of the inhibition zones were expressed and measured in mm. Each assay was repeated three times in this experiment, and the results were expressed as a mean value ± standard deviation.

#### 2.3.3. Minimum Inhibitory (MIC) and Minimum Fungicidal Concentration (MFC)

The MICs and MFCs were carried out as described by Rasadah and Muharnad [32]. In this regard, the nutrient agar and Sabouraud agar medium were inoculated with freshly prepared cells of *C. albicans*. The discs were dipped into the DMSO containing each compound and placed on the plates at concentration ranging between 50 and 300 μg per disc. Next, the discs were placed gently onto the surface of the Petri dishes after the bacterial growth and evaporation of DMSO (where it was used as a negative control). After the incubation of the Petri dishes at 37 °C for 24 h, antifungal activity was measured. Each assay was repeated three times in this experiment, and the results were expressed as a mean value.

#### 2.3.4. Methodology of Docking Studies

In the present study, the crystal structure of *N*-myristoltransferase (NMT) was retrieved under the accession code 1IYL [33]. The protein structure was prepared using the protein preparation wizard in Molecular Operating Environment (MOE) Version 2018.0101 [34]. During preparation, the structures were subjected to addition of missing atoms and residues, bond order, formal charge correction, and tautomer adjustment. Hydrogens atoms were added using the protonate 3D algorithm. For protonation, GB/VI was used as the electrostatics function, with a dielectric value of 80 (for solvent). The van der Waals and electrostatics cutoffs were set at 15 and 10 A, respectively. Partial charges were applied using the AMBER10: EHT force field. The water molecules involved in the bridging ligand and protein were retained and kept rigid during energy minimization. The ligands were charged and minimized using the MMFF94x force field in MOE. The graphics were obtained using the MOE software suite.

## 3. Results and Discussion

### 3.1. Chemistry

**Scheme 1:**
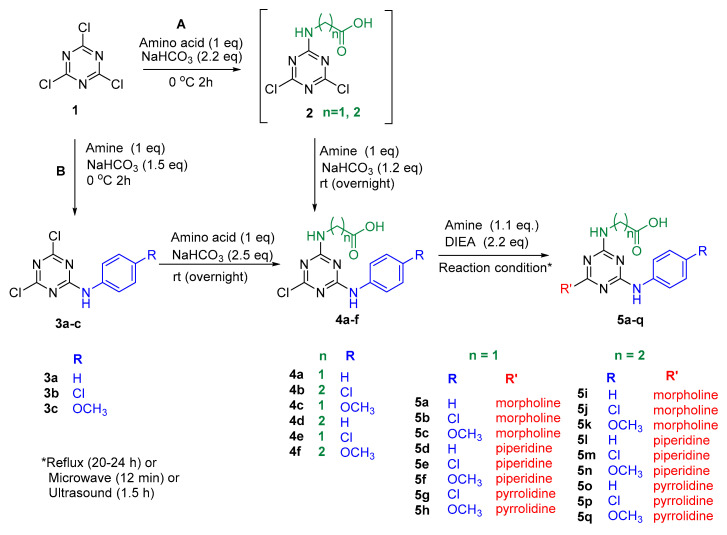
Synthesis of di- and tri-substituted *s*-triazine amino acid derivatives.

Disubstituted *s*-triazinyl amino acid derivatives (**5a–q**) were prepared as demonstrated in Scheme 1 via either a one-pot synthesis (Route A) or stepwise synthetic pathway (Route B). In the one-pot synthesis, the amino acid was added to cyanuric chloride at 0 °C using aq. NaHCO_3_ as base. After 2 h, substituted aniline derivatives were added in the presence of aq. NaHCO_3_ as base, and the reaction was stirred overnight at rt to afford **4a–f**. In contrast, the stepwise strategy (Route B, Scheme 1) involved the addition of substituted aniline derivative to cyanuric chloride at 0 °C for 2 h using NaHCO_3_ as base to form **3a–c**. After isolation of **3a–c**, underwent a nucleophilic substitution reaction with different amino acids to afford the products **4a–f**.

No marked difference in the yield of **4a–f** between the two routes (A and B) was observed. However, the stepwise reaction decreased the probability of side product formation from the reagents remaining in the one-pot reaction. It is important to highlight that sufficient amount of NaHCO_3_ was used during the synthesis to overcome/neutralize traces of cyanuric acid, which might be present in the crude cyanuric chloride.

The synthesis of **5a–q** required elevated temperatures, which were achieved in three ways, namely by conventional heating, microwave irradiation or ultrasonication. Disubstituted *s*-triazine derivatives **4a–f** (1 equiv.) and secondary amines (1.1 equiv.) were reacted in the presence of diisopropylethylamine (DIEA) as a base and THF as solvent in the three conditions mentioned above. Using conventional heating (reflux for 20–24h), **5a–q** were obtained in good yields and purities. In contrast, the use of microwave irradiation afforded these compounds in better yields in only 12–15 min of reaction time, while the application of ultrasonic irradiation allowed their synthesis at room temperature in 1.5h with a greater yield and purity than conventional heating (Table 1). Of note, the prolonged reaction under conventional heating can be markedly reduced by means of microwave or ultrasound irradiation. The structures of all compounds **5a–q** were confirmed by NMR spectra (^1^H and ^13^C) as shown in Supporting information (Figures S1–S17).

### 3.2. In Vitro Antimicrobial Assays

The antibacterial and antifungal activity of **5a–q** was tested. None of the tested compounds showed antibacterial activity against the two Gram-positive bacteria (*S. aureus* and *S. epidermidis*) or two Gram-negative bacteria (*E. coli* and *S. typhimurium*). However, they showed promising antifungal activity against the human pathogenic *C. albicans* compared with the reference standard drug clotrimazole (Table 2). The effective bioactivity of 4,6-disubstituted s-triazin-2-yl amino acid derivatives with fungal strain, and no activity on bacterial strains may be dependent on several features of both species like cell wall, cell membrane or metabolic activity and the different amino acid moiety used for the preparation of these derivatives [35]. In addition, the proteinaceous transfer system for these bioactive compounds, primarily in the fungal strain, is responsible for the process [36].

**Figure 1:**
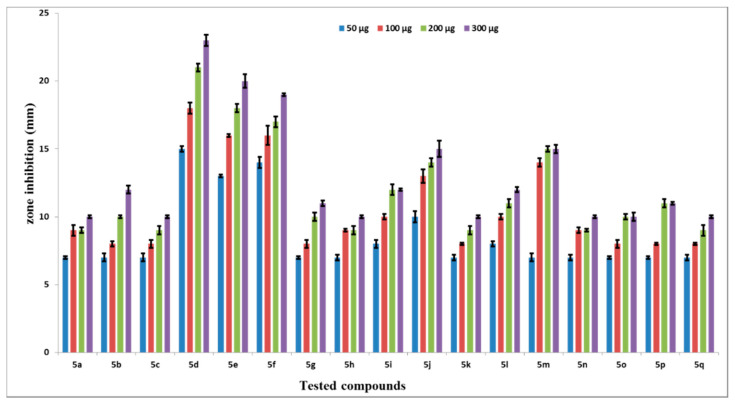
Inhibition zones of **5a–q** at concentrations of 50,100, 200 and 300 μg per disc against *C. albicans*. Error bars mean the STDV of the average values of the triplicate experiments.

The growth-inhibiting effects of **5a–q** against *C. albicans* were quantitatively determined by means of the disc diffusion method at concentrations of 50, 100, 200, and 300 μg per disc. The results are shown in Table 2 and Figure 1. Compounds **5d**, **5e**, and **5f** showed the best inhibitory capacity at 50 μg per disc of 15, 13, and 14 mm, respectively. These results revealed that the presence of the piperidine is crucial for the activity. Thus, the combination of piperidine with aniline derivatives on the triazine core bearing the glycine amino acid **5d** gave the best results. In addition, among the aniline derivatives, chloro appeared to be more active than methoxy. These results are consistent with the notion that compounds bearing electron-withdrawing groups, such as chlorine atoms, have a potent inhibitory effect on the examined microbe [19,37]. This observation was also noticed in all tested compounds at different concentrations, as shown in Figure 1.

The individual minimum inhibitory concentration (MIC, μM) and minimum fungicidal concentration (MFC, μM) values of **5a–q** against *C. albicans* are listed in Table 2. In general, all the compounds showed good antifungal activities, with a MIC value falling between 33.47 and 37.95 μM and MFC value between 66.58 and 75.90 μM. Again, this assay supports the previous findings illustrated inTable 2.

### 3.3. Docking Studies

**Figure 2:**
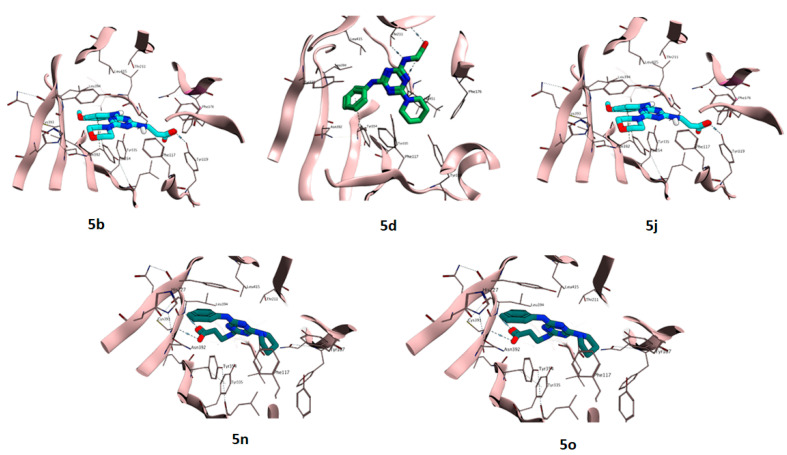
The protein–ligand interaction profile of the ligands (**5b**, **5d**, **5j**, **5n**, and **5o**).

The observed antifungal activity of the compounds can be attributed to the inhibition of *N*-myristoltransferase (NMT) enzyme, which has been validated as an anti-fungal target [32]. NMT activity is essential for the vegetative growth of *C. albicans*. NMT is a promising target enzyme for the development of novel fungicidal drugs with a broad anti-fungal spectrum. To date, various types of NMT inhibitors have been reported, including peptidomimetics, benzothiazoles, tetrahydro- carbazoles, sulphonamides and benzofurans. Recently, we have carried out an extensive virtual screening to identify NMT inhibitors effective against kinetoplastids. Therefore, we examined the protein–ligand interaction profile of **5b**, **5d**, **5j**, **5n** and **5o** with NMT. The docking profile of compound **5d** suggests that the compound is well accommodated in the binding site of *C. albican*s NMT. The *s*-triazine moiety mediates hydrogen bonds with the side chain hydroxyl of Y225. The Y225 also mediates another hydrogen bond with ligand **5o** (Figure 2). The benzene ring of Y225 exhibits pi stacking interaction with the benzene ring of the ligands. Furthermore, the hydrogen bonding between the ligand and backbone amide of L355 and L394 provides anchorage. Moreover, the ligand also demonstrates hydrophobic contact with surrounding residues, which explains the inhibitory potential of **5o** observed.

A slightly different binding mode was observed in the triazine moiety of **5d** mediate hydrogen bond interaction with L451 (Figure 2). Y225 exhibits pi stacking interaction with the benzene ring. The compound also demonstrates polar contact with the side chain of T211. An extensive network of hydrogen and hydrophobic contacts provides further anchorage.

The protein–ligand interaction profile of **5j**, **5b** and **5n** demonstrated that the triazine and benzene moieties of the ligands are stacked against Y359 (Figure 2). The methoxy substitution shows polar contact with L394. However, the oxygen atom of the oxathiane ring mediates the hydrogen bond with N392.

Our results demonstrate that all the compounds are well accommodated and exhibit complementarity with the active site residues of *C. albicans* NMT, which explains the observed antifungal activity.

**Figure 3:**
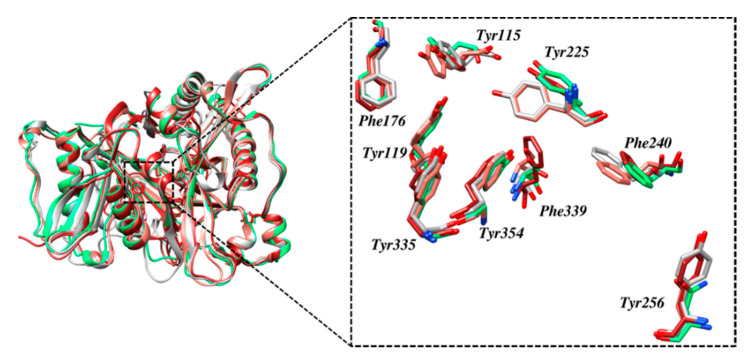
Alignment of active site of *C. albicans N*-myristoltransferase (NMT) (firebrick color) with *C. neoformans* NMT (grey), *Aspergillus fumigatus* (spring green color) NMT and *Histoplasma capsulatum* NMT (salmon color).

Furthermore, to explore the antifungal activity against other pathogenic fungi, the active site of *Cryptococcus neoformans, Aspergillus fumigatus* and *Histoplasma capsulatum* NMTs were aligned to *C. albicans* NMT, as known crystal structures of NMTs from different fungi indicate that all NMTs have similar substrate binding sites despite their low sequence identity. The sequence similarity is obtained from blastp between *C. albicans* NMT with *C. neoformans* NMt (46%), *A. fumigatus* NMT (49%) and *H. capsulatum* NMT (46%), and, interestingly, they share a similar substrate binding site. As depicted in Figure 3, the alignment of the active sites of NMTs from different fungi reveals that all the active site residues in *C. albicans* were well aligned with *C. neoformans*, *A. fumigatus* and *H. capsulatum*. However, Tyr256 in *C. albicans* replaced Asn249 and Asn331 in *A. fumigatus* and *H. capsulatum,* respectively, while Phe339 in *C. albicans* replaced Ser378, Ser351 and Ser415 in *A. fumigatus*, *C. neoformans* and *H. capsulatum,* respectively. This high degree of similarity between binding sites may be attributed to the broad spectrum antifungal activities, and these compounds could be active against other pathogenic fungi species.

## 4. Conclusions

Novel 4,6-disubstituted s-triazin-2-yl amino acid derivatives were prepared via conventional heating, microwave irradiation and ultrasonication. The two latter techniques gave **5a–q** in good purity and better yield within shorter time intervals compared to the conventional heating method. The synthesized derivatives were not found to be active against two Gram-positive bacteria (*S. aureus ATCC* 29213*, S. epidermidis ATCC 12228*) and two Gram-negative bacteria (*E. coli ATCC 25922 and S. typhimurium ATCC 14028*). However, they showed promising antifungal activities against the human pathogen *C. albicans* (*ATCC 60193*).

The key to the antifungal activity is the presence of the piperidine ring. Thus, the compounds containing piperidine were the most active, which agreed with the previously reported data [29]. Interestingly, morpholine (six-membered ring containing one O instead of C as in the case of piperidine) and pyrrolidine (five-membered ring) showed less activity. This supports the need for a degree of hydrophobicity in this position.

On the other hand, aniline without any substituent proved to be more promising than chloro (electron-withdrawing) and methoxy (electron-donating) analogs. In addition, the synthesized compounds were found to be well accommodated in the active site residues of *C. albicans* NMT, which explains the observed antifungal activities.

The known crystal structures of NMTs from different pathogenic fungi, namely, *C. neoformans*, *A.* fumigatus and *H. capsulatum,* indicate that all NMTs share similar substrate binding sites. This high degree of similarity between binding sites may be attributed to the broad-spectrum antifungal activities, and these compounds could be active against other pathogenic fungi species.

**Figure 4:**
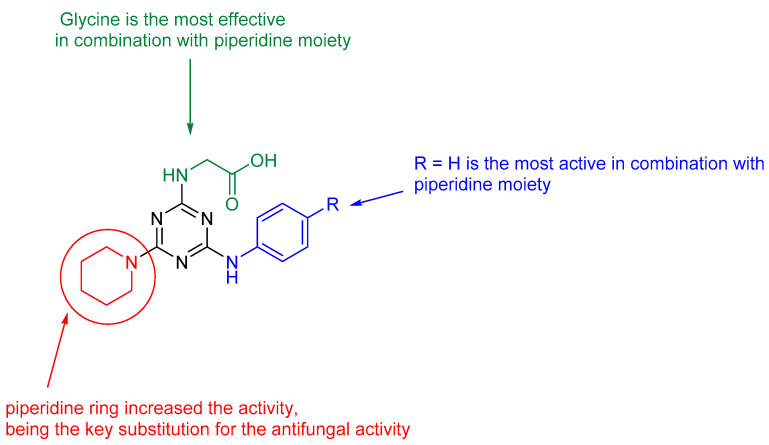
The structure–activity relationship.

Finally, we can summarize the structure–activity relationship of our target compounds as indicated in Figure 4.

To gain a complete picture of the effect of structural modification or functionalization on antifungal activities, further development for a new series based on *s*-triazine amino acid derivatives with different substituents will be considered in our laboratories.

## Figures and Tables

**Table 1:** Yield (%) of the synthesis of **5a–q** derivatives via conventional heating, microwave irradiation and ultrasonic methods.

Compound	Conventional (20–24 h)	Microwave (12–15 min)	Ultrasound (1.5 h)
**5a**	89	91	92
**5b**	87	90	92
**5c**	86	90	90
**5d**	89	93	92
**5e**	88	91	92
**5f**	87	90	90
**5g**	86	92	91
**5h**	86	90	93
**5i**	84	91	93
**5j**	86	93	90
**5k**	88	90	92
**5l**	89	92	94
**5m**	86	92	92
**5n**	89	94	93
**5o**	87	91	92
**5p**	89	92	90
**5q**	86	90	92

**Table 2:** Zone of inhibition (mm) *, Minimum Inhibitory Concentration (MIC) and Minimum Fungicidal Concentration (MFC) of the target compounds against *Candida albicans* growth.

Compound	*C. albicans* (mm)				MIC (μM)	MFC (μM)
	50 µg	100 µg	200 µg	300 µg		
**5a**	7 ± 0.1	9 ± 0.4	9 ± 0.2	10 ± 0.1	37.76 ± 0.7	75.52 ± 0.7
**5b**	7 ± 0.3	8 ± 0.2	10 ± 0.1	12 ± 0.3	34.14 ± 0.1	68.28 ± 0.1
**5c**	7 ± 0.3	8 ± 0.3	9 ± 0.3	10 ± 0.1	34.58 ± 0.8	69.16 ± 0.7
**5d**	15 ± 0.2	18 ± 0.4	21 ± 0.3	23 ± 0.4	37.95 ± 0.2	75.90 ± 0.9
**5e**	13 ± 0.1	16 ± 0.1	18 ± 0.3	20 ± 0.5	34.36 ± 0.6	68.72 ± 0.8
**5f**	14 ± 0.4	16 ± 0.7	17 ± 0.4	19 ± 0.1	34.74 ± 0.3	69.48 ± 0.3
**5g**	7 ± 0.1	8 ± 0.3	10 ± 0.3	11 ± 0.2	35.73 ± 0.7	71.46 ± 0.5
**5h**	7 ± 0.2	9 ± 0.1	9 ± 0.3	10 ± 0.1	36.19 ± 0.2	72.38 ± 1.0
**5i**	8 ± 0.3	10 ± 0.2	12 ± 0.4	12 ± 0.1	36.19 ± 0.4	72.38 ± 0.9
**5j**	10 ± 0.4	13 ± 0.5	14 ± 0.3	15 ± 0.6	33.29 ± 0.3	66.58 ± 0.6
**5k**	7 ± 0.2	8 ± 0.1	9 ± 0.3	10 ± 0.1	36.19 ± 0.2	72.38 ± 1.2
**5l**	8 ± 0.2	10 ± 0.2	11 ± 0.3	12 ± 0.2	36.40 ± 0.3	72.8 ± 0.4
**5m**	7 ± 0.3	14 ± 0.3	15 ± 0.2	15 ± 0.3	33.09 ± 0.1	66.18 ± 0.9
**5n**	7 ± 0.2	9 ± 0.2	9 ± 0.1	10 ± 0.1	33.47 ± 0.5	66.94 ± 1.2
**5o**	7 ± 0.1	8 ± 0.3	10 ± 0.2	10 ± 0.3	37.95 ± 0.7	75.90 ± 0.9
**5p**	7 ± 0.1	8 ± 0.1	11 ± 0.3	11 ± 0.1	34.35 ± 0.4	68.70 ± 0.6
**5q**	7 ± 0.2	8 ± 0.1	9 ± 0.4	10 ± 0.1	34.78 ± 0.6	69.56 ± 0.4
Clotrimazole **	-	-	-	23 ± 0.2	-	-

* Inhibition zone (mm) at 300 µg per disc; ** 100 µg per disc, ± means Standard deviation (STDV) of triplicate experiments.

## Supplementary Materials

The following are available online at https://www.mdpi.com/2309-608X/6/4/237/s1, Figures S1–S17: ^1^H-NMR and ^13^C-NMR for compound **5a–q**.
