# Supplementary material for: Novel 4,6-Disubstituted s-Triazin-2-yl Amino Acid Derivatives as Promising Antifungal Agents
Source: J Fungi (Basel). 2020 Oct 21;6(4):237. doi: 10.3390/jof6040237 (PMC7712924; doi:10.3390/jof6040237)

Supporting Information

Novel 4,6-Disubstituted *s*-Triazin-2-yl Amino Acid Derivatives as Promising Antifungal Agents

Rakia Abd Alhameed, Zainab Almarhoon, Essam N. Sholkamy, Salman Ali Khan, **Zaheer Ul-Haq, Anamika Sharma, Beatriz G. de la Torre, Fernando Albericio,* Ayman El-Faham***

Table of Content

**Figure S1**	^{1}H-NMR and ^{13}C-NMR for compound **5a**
**Figure S2**	^{1}H-NMR and ^{13}C-NMR for compound **5b**
**Figure S3**	^{1}H-NMR and ^{13}C-NMR for compound **5c**
**Figure S4**	^{1}H-NMR and ^{13}C-NMR for compound **5d**
**Figure S5**	^{1}H-NMR and ^{13}C-NMR for compound **5e**
**Figure S6**	^{1}H-NMR and ^{13}C-NMR for compound **5f**
**Figure S7**	^{1}H-NMR and ^{13}C-NMR for compound **5g**
**Figure S8**	^{1}H-NMR and ^{13}C-NMR for compound **5h**
**Figure S9**	^{1}H-NMR and ^{13}C-NMR for compound **5i**
**Figure S10**	^{1}H-NMR and ^{13}C-NMR for compound **5j**
**Figure S11**	^{1}H-NMR and ^{13}C-NMR for compound **5k**
**Figure S12**	^{1}H-NMR and ^{13}C-NMR for compound **5l**
**Figure S13**	^{1}H-NMR and ^{13}C-NMR for compound **5m**
**Figure S14**	^{1}H-NMR and ^{13}C-NMR for compound **5n**
**Figure S15**	^{1}H-NMR and ^{13}C-NMR for compound **5o**
**Figure S16**	^{1}H-NMR and ^{13}C-NMR for compound **5p**
**Figure S17**	^{1}H-NMR and ^{13}C-NMR for compound **5q**

Figure S1 ^{1}H-NMR and ^{13}C-NMR for compound **5a**

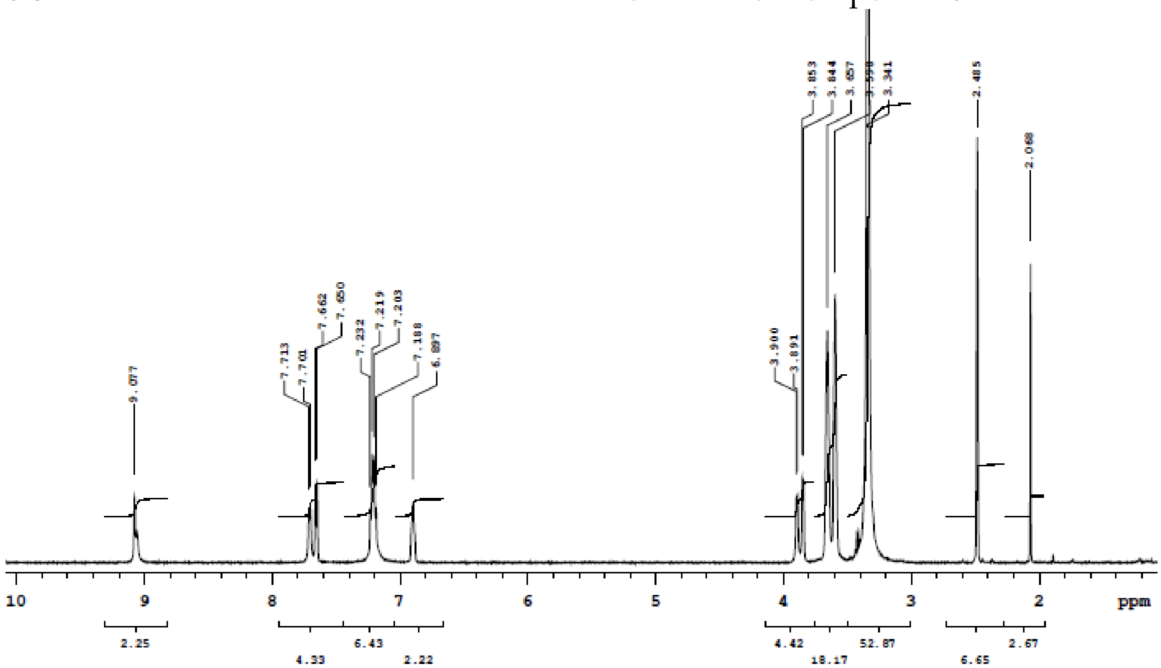

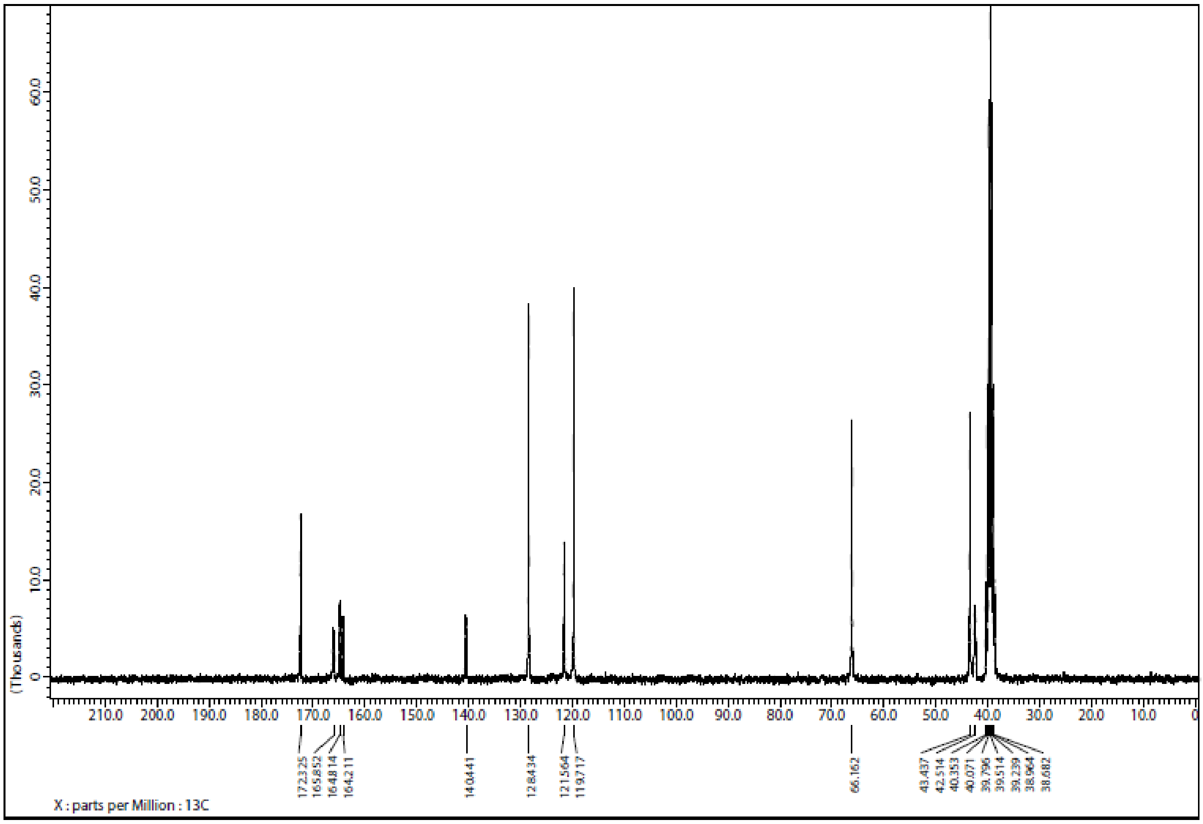

Figure S2 ^{1}H-NMR and ^{13}C-NMR for compound **5b**

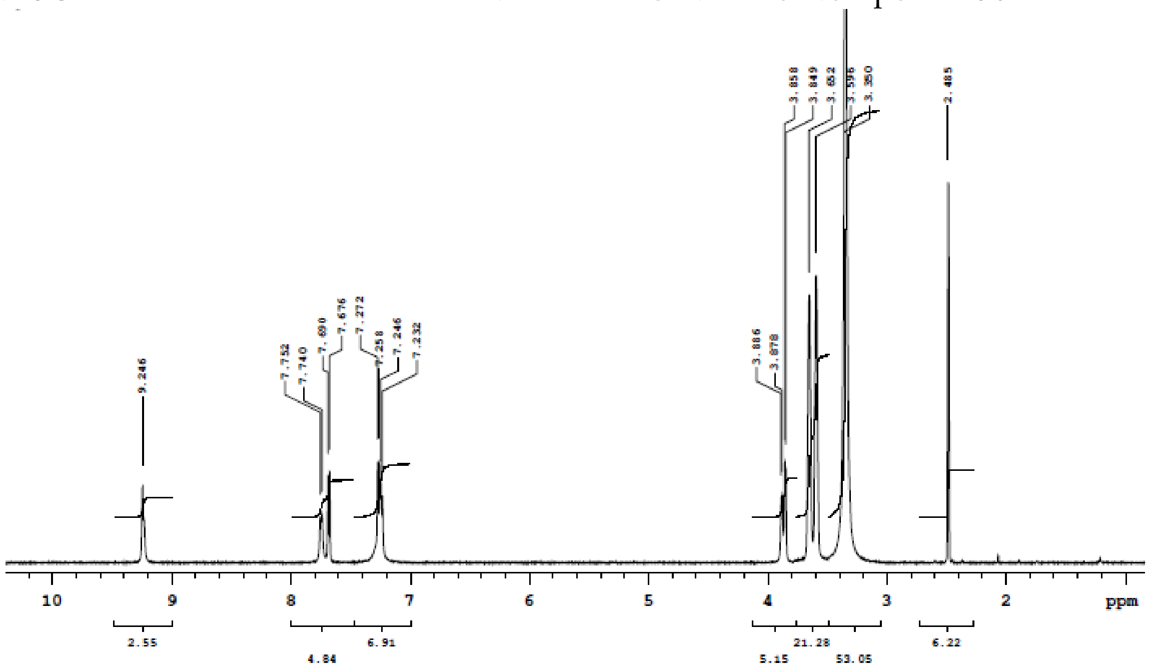

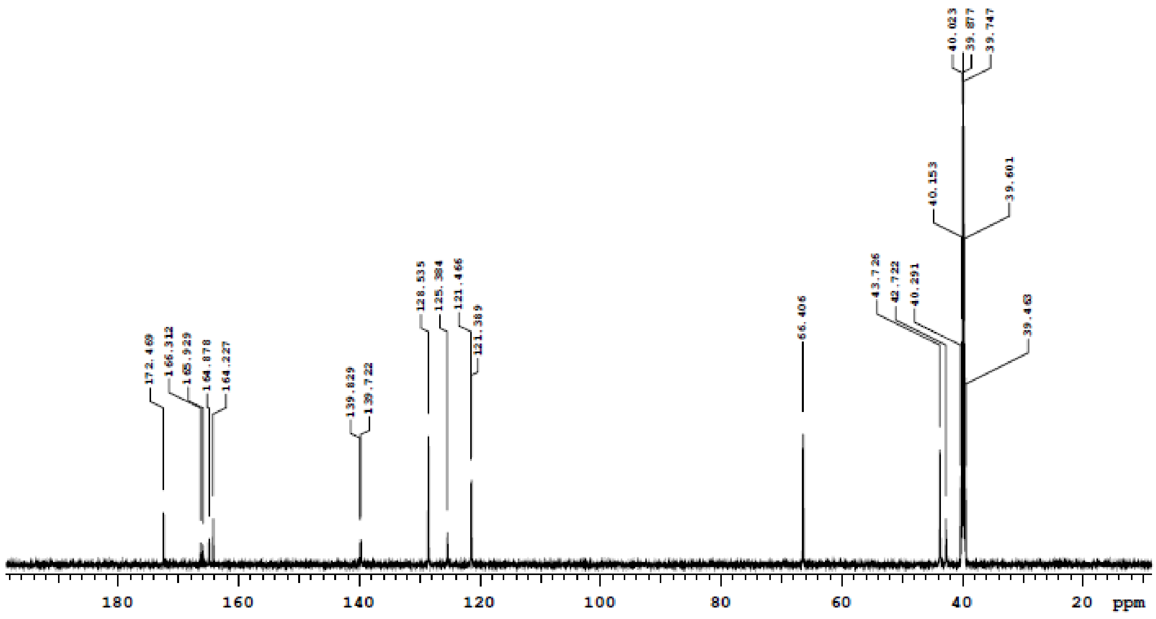

Figure S3 ¹H-NMR and ¹³C-NMR for compound **5c**

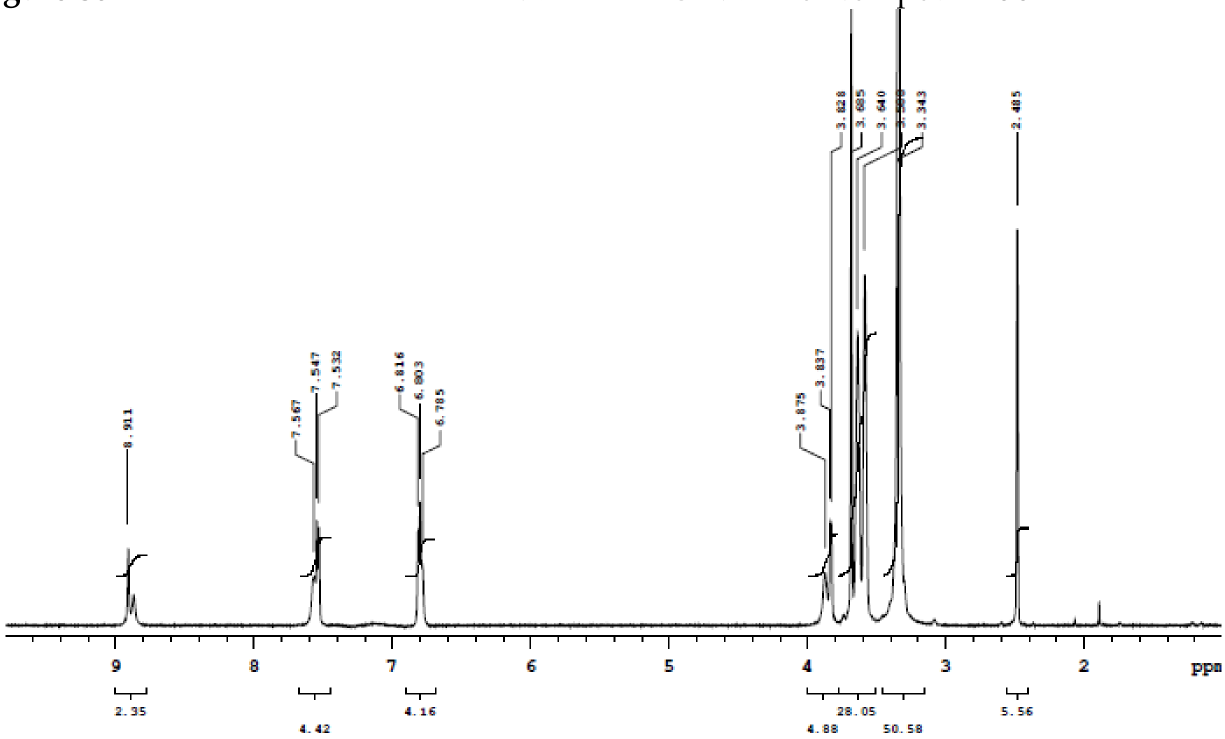

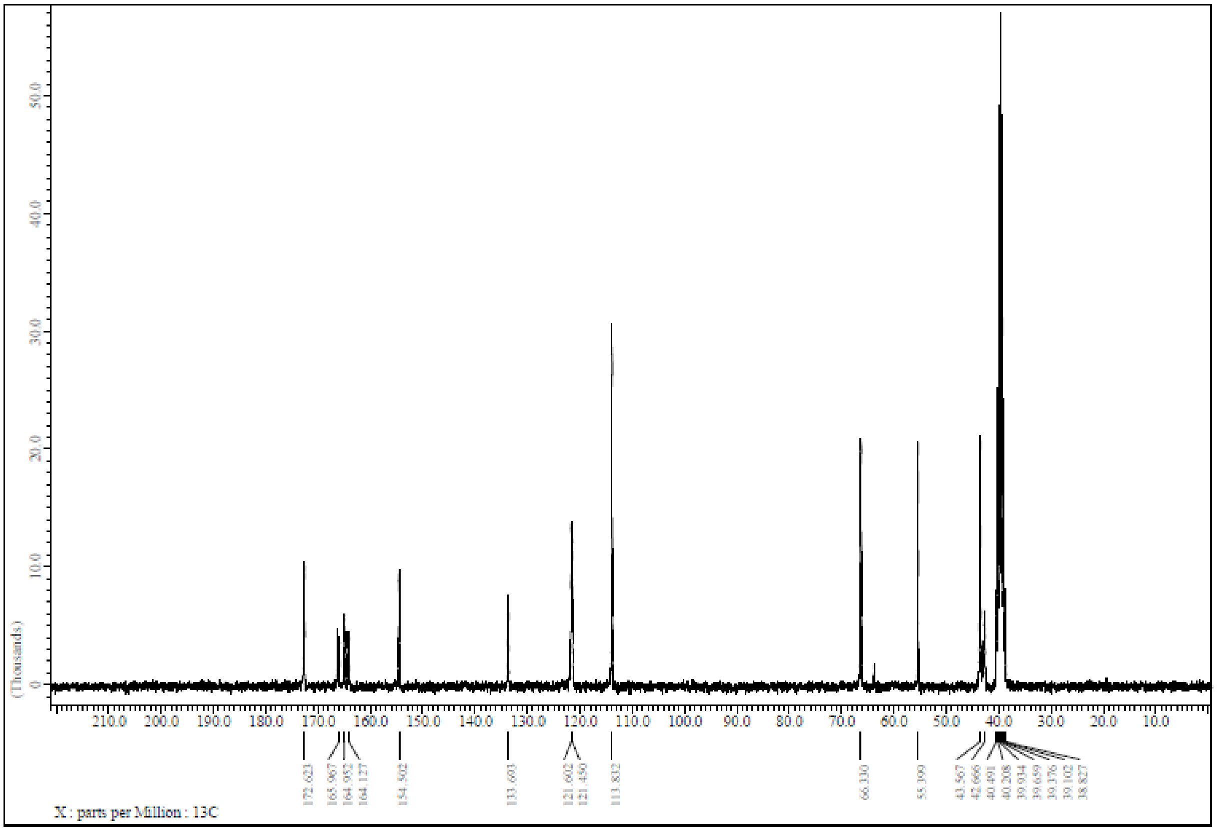

Figure S4 ^{1}H-NMR and ^{13}C-NMR for compound **5d**

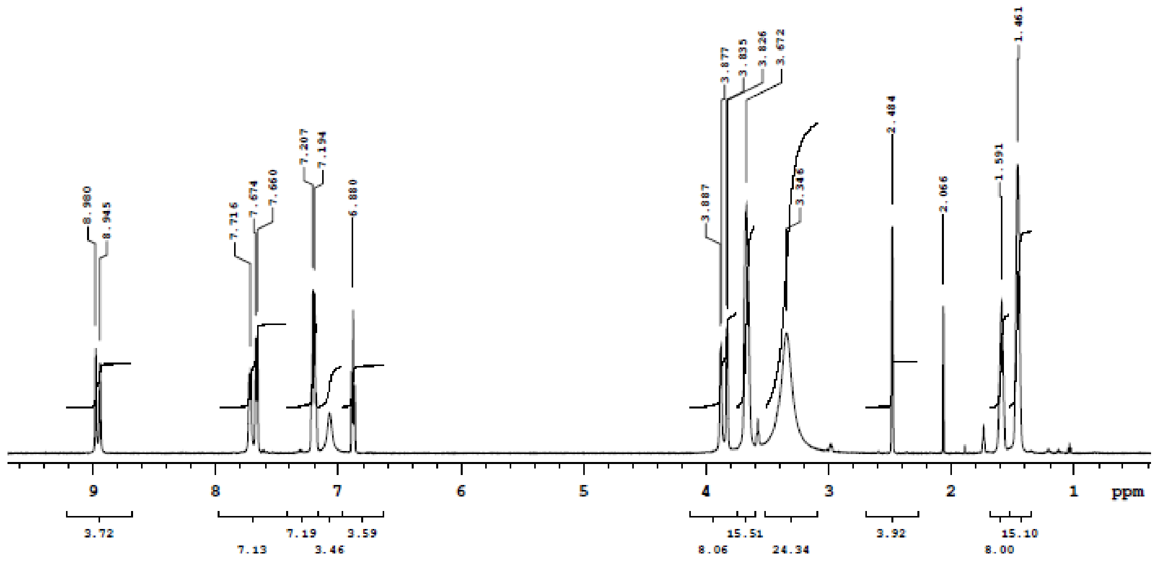

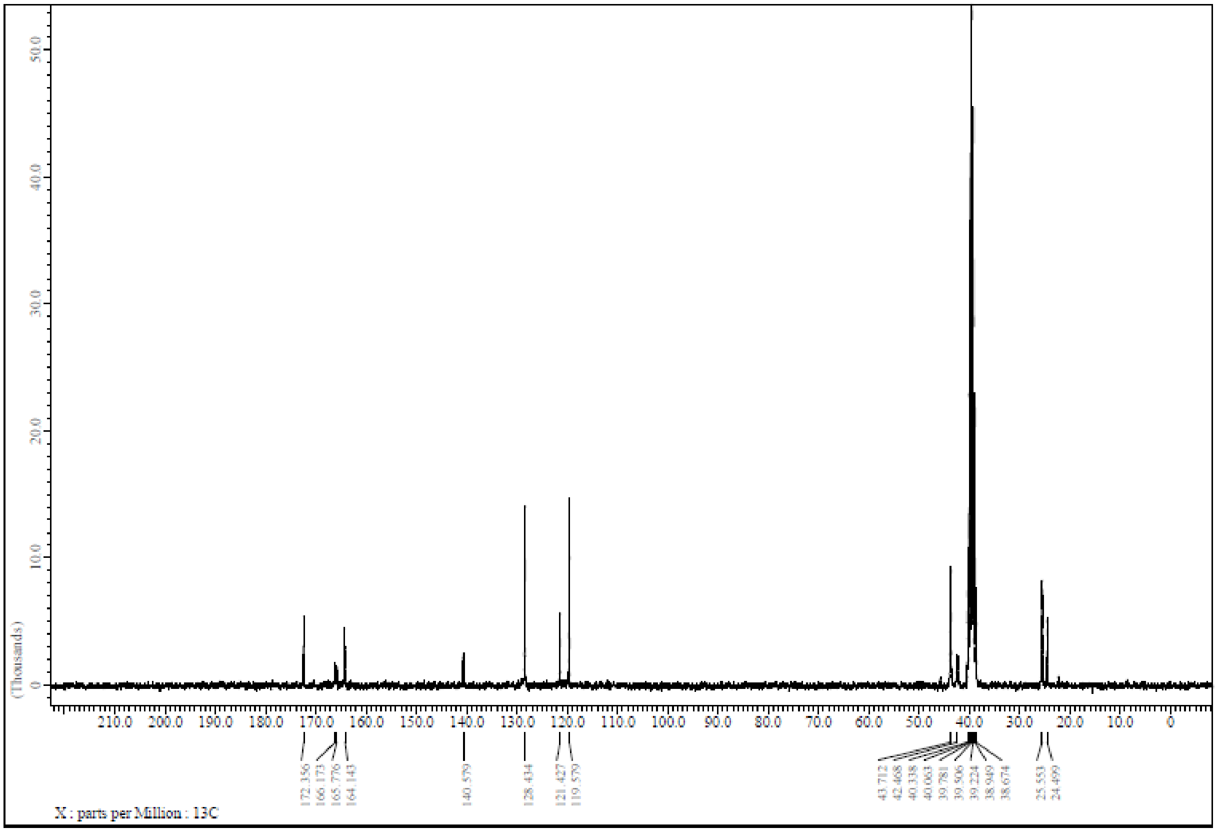

Figure S5 ^{1}H-NMR and ^{13}C-NMR for compound **5e**

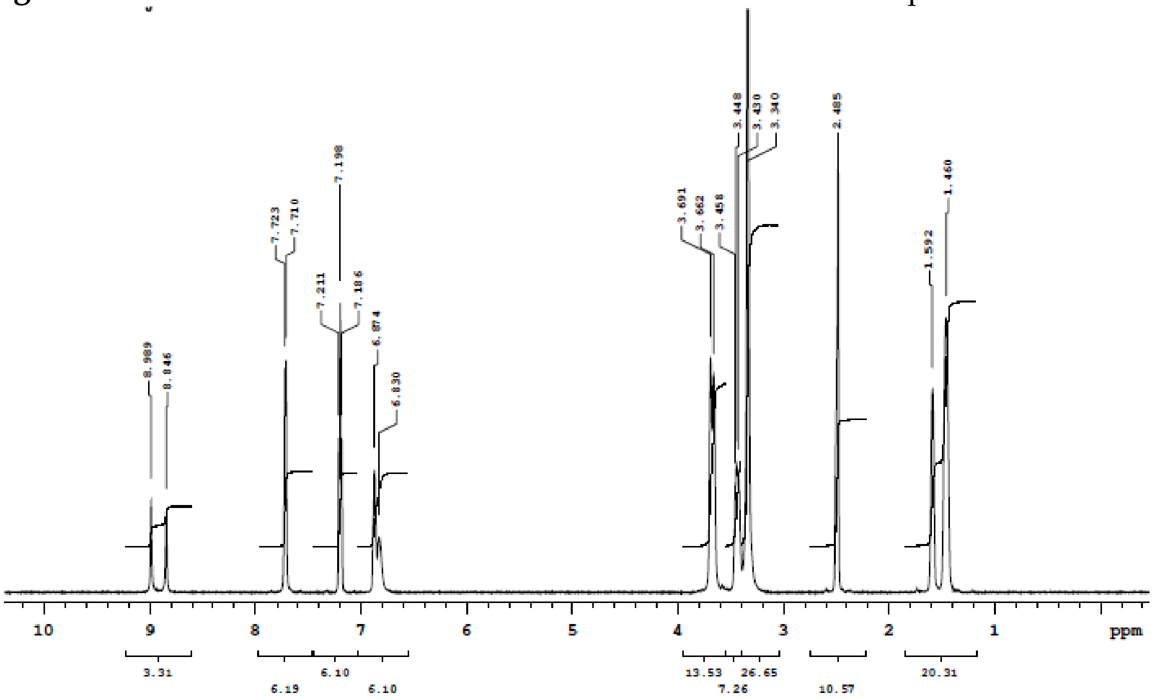

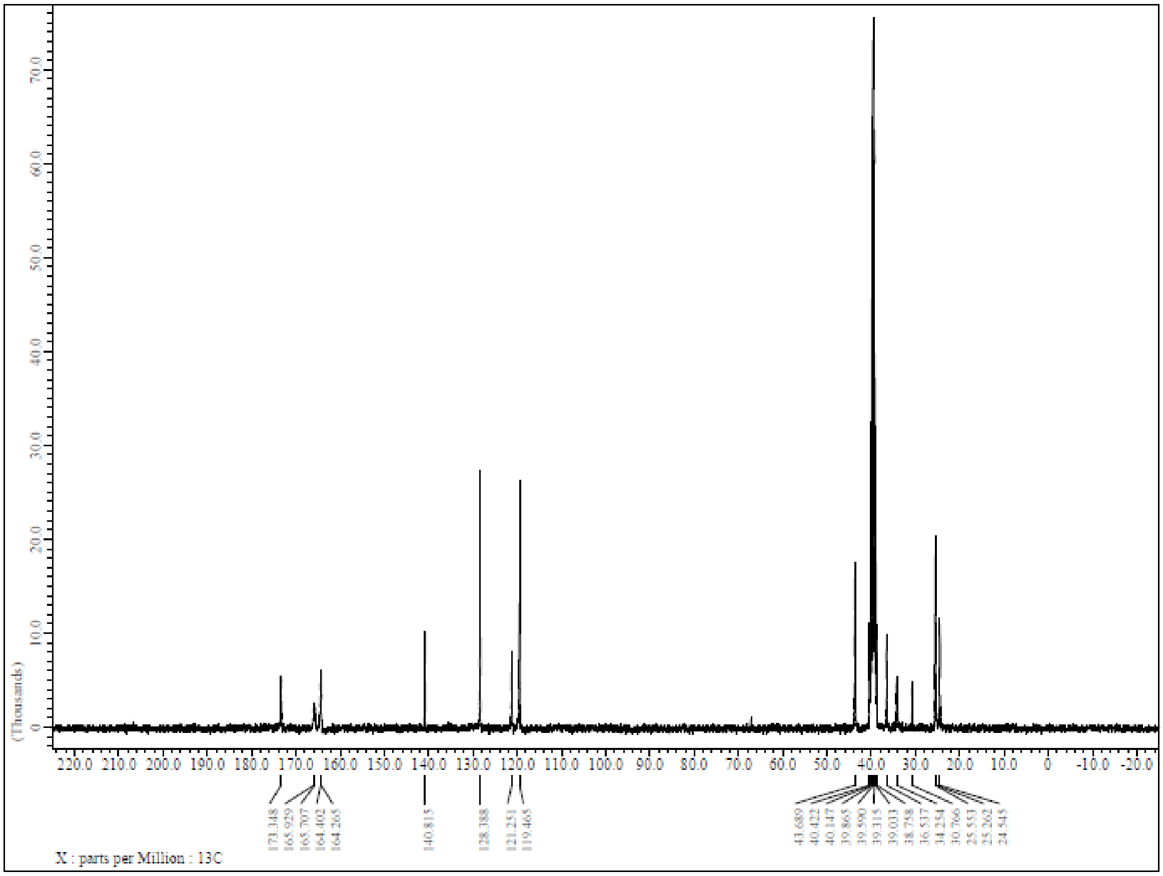

Figure S6 ^{1}H-NMR and ^{13}C-NMR for compound **5f**

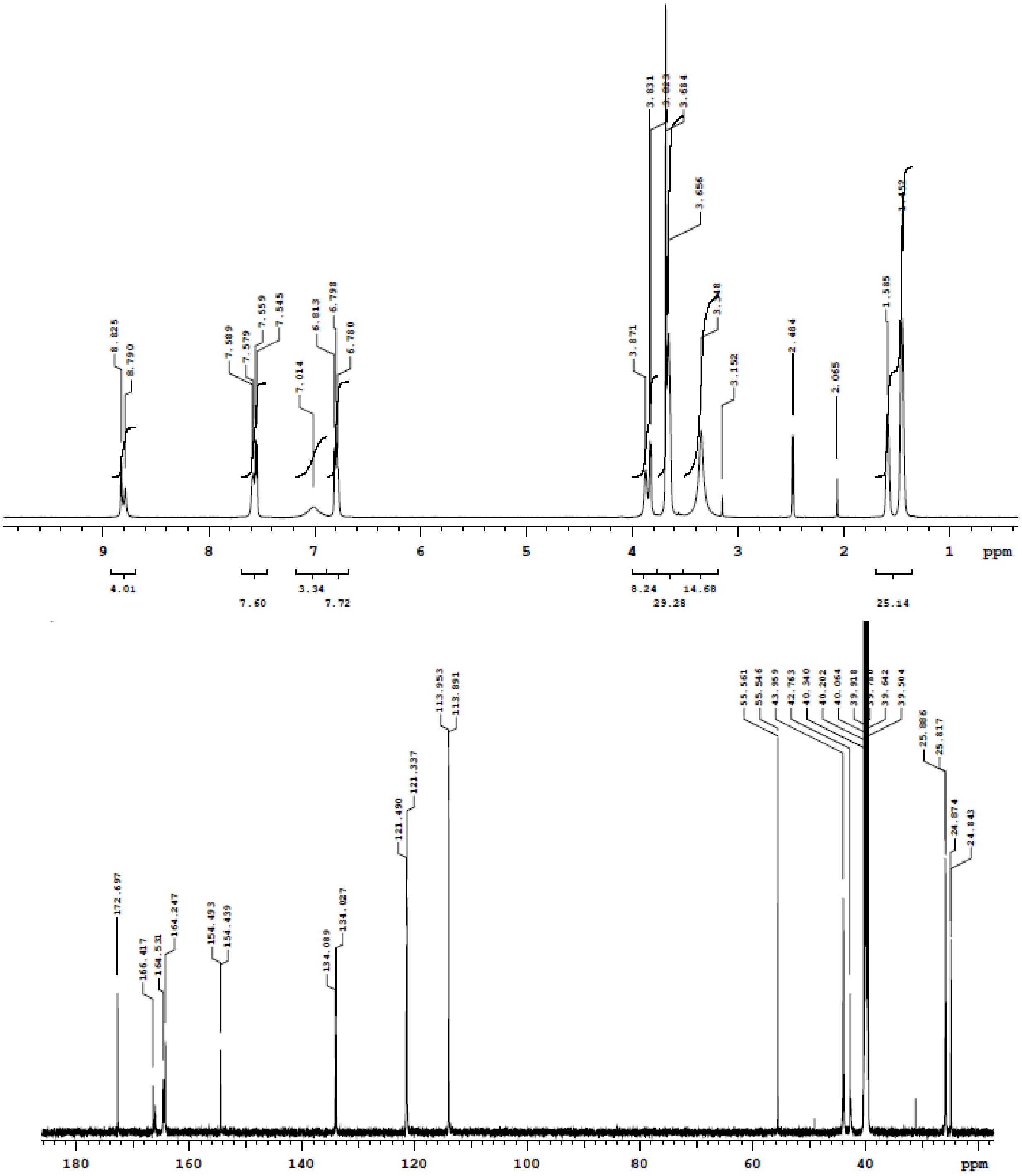

Figure S7 ^{1}H-NMR and ^{13}C-NMR for compound **5g**

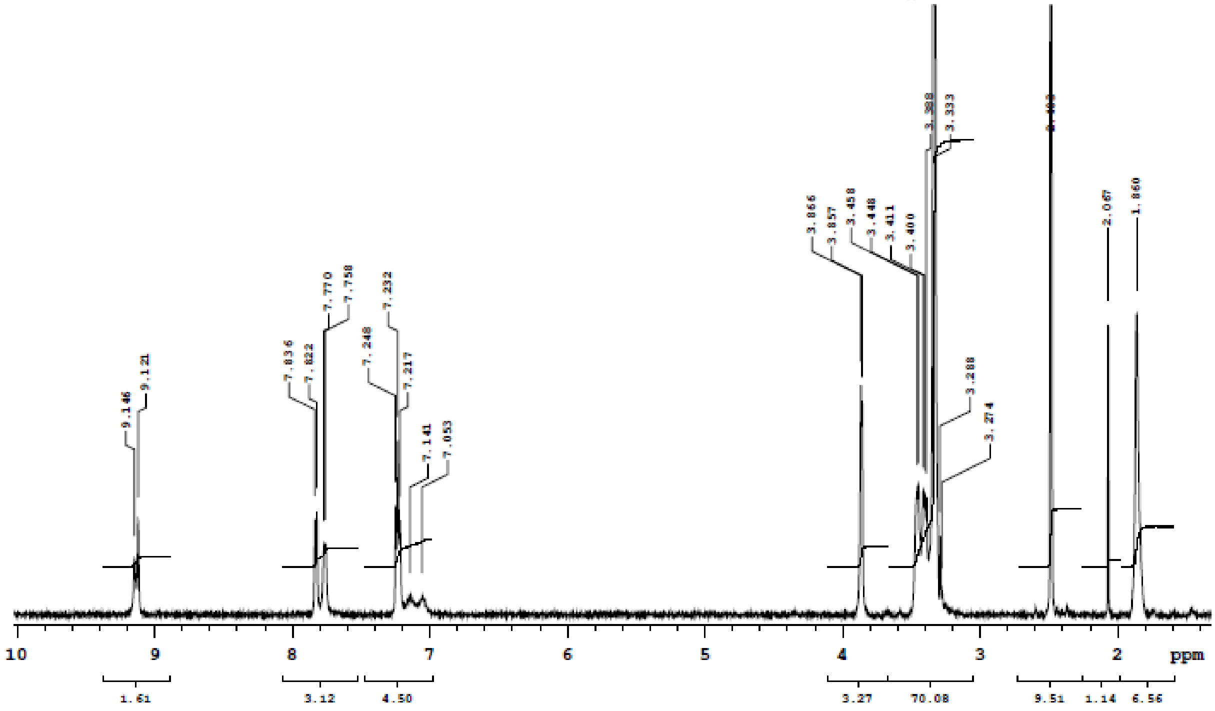

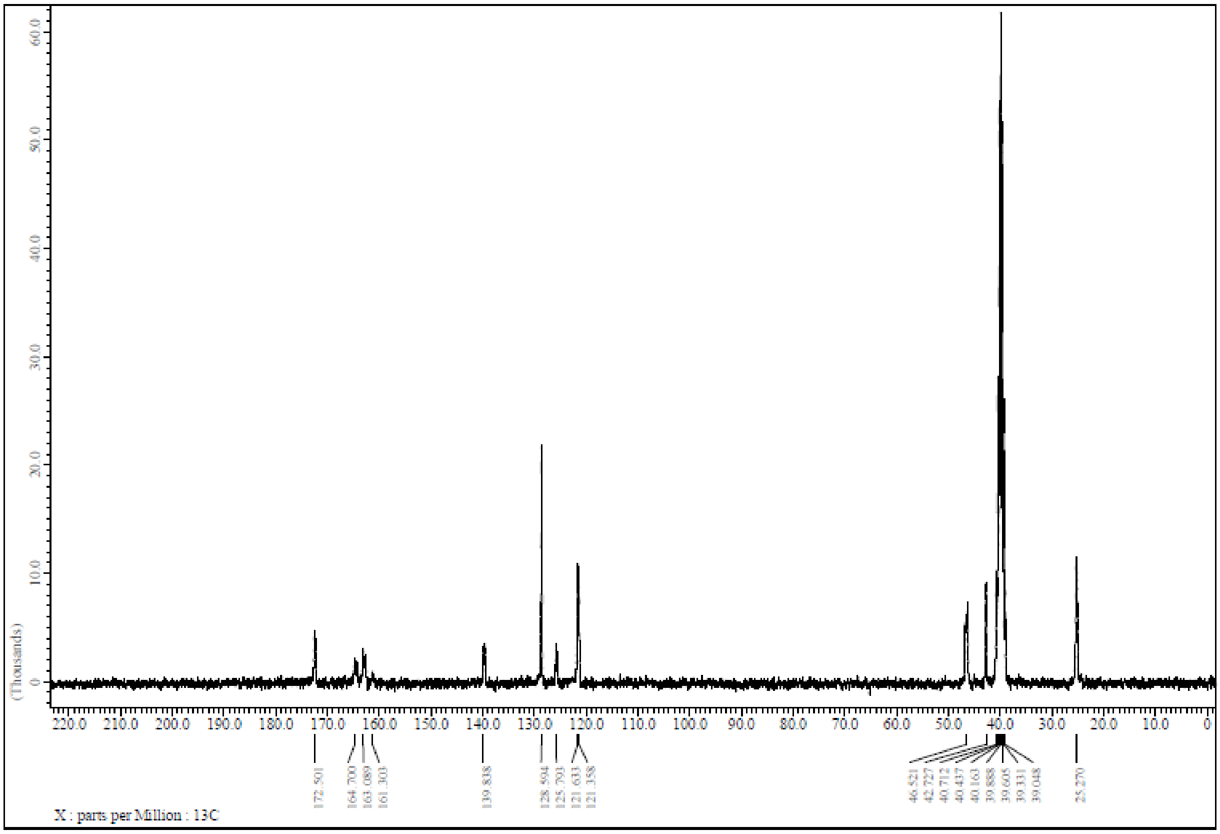

Figure S8 ^{1}H-NMR and ^{13}C-NMR for compound **5h**

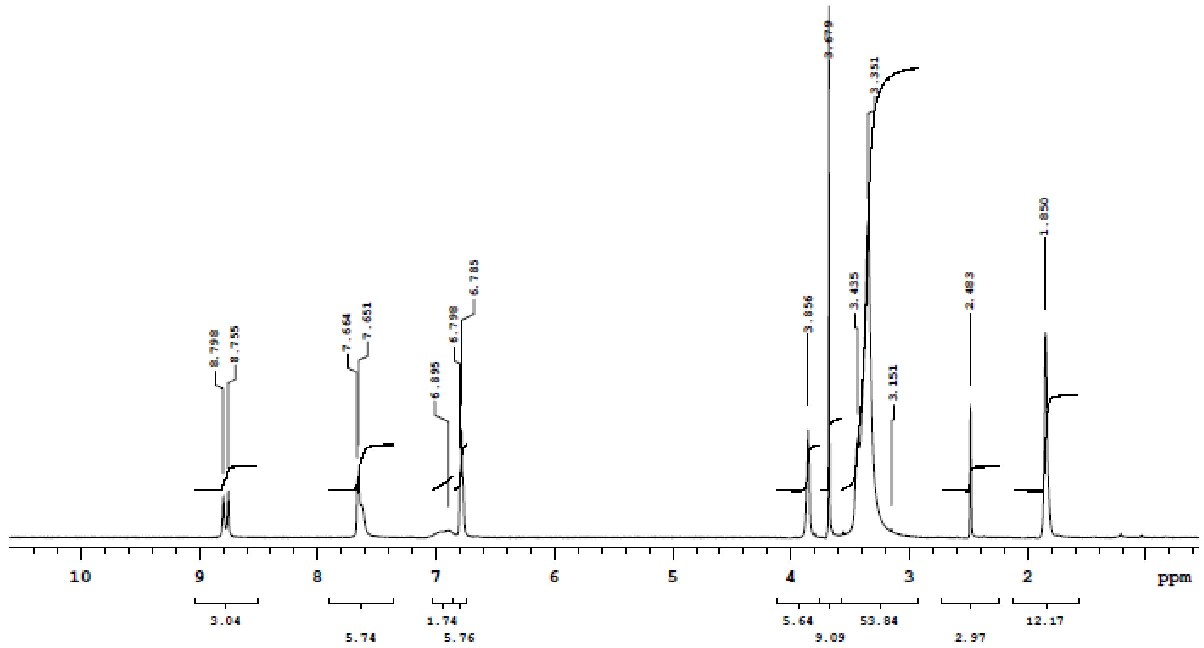

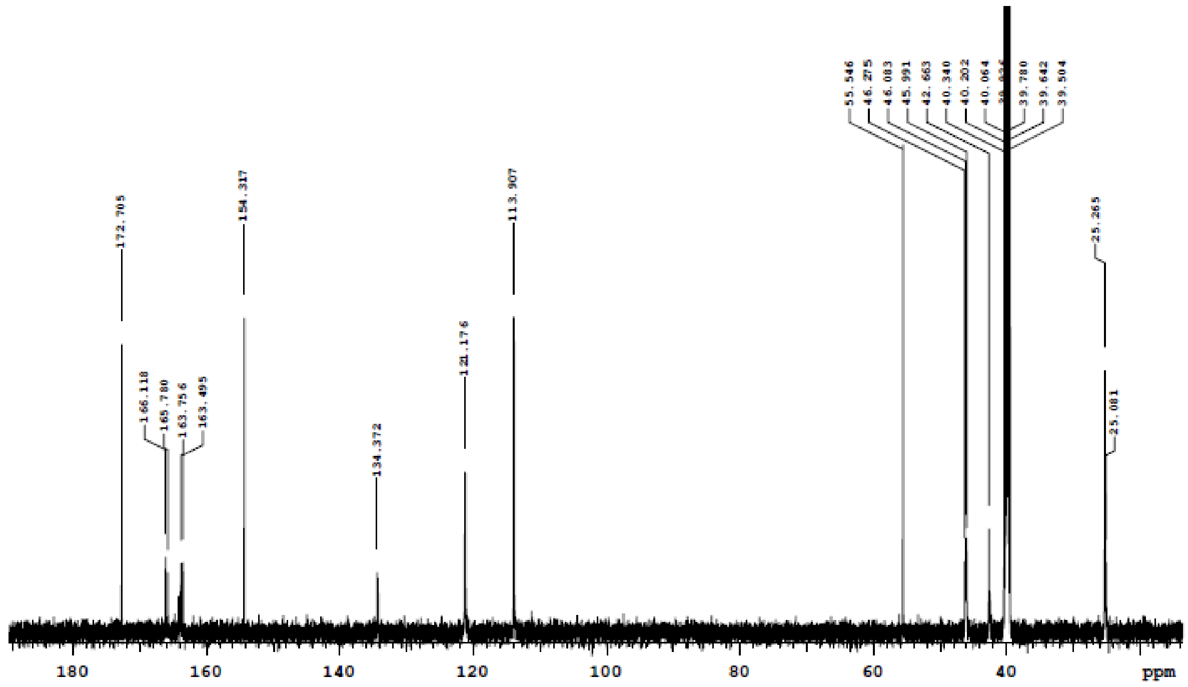

Figure S9 ^{1}H-NMR and ^{13}C-NMR for compound **5i**

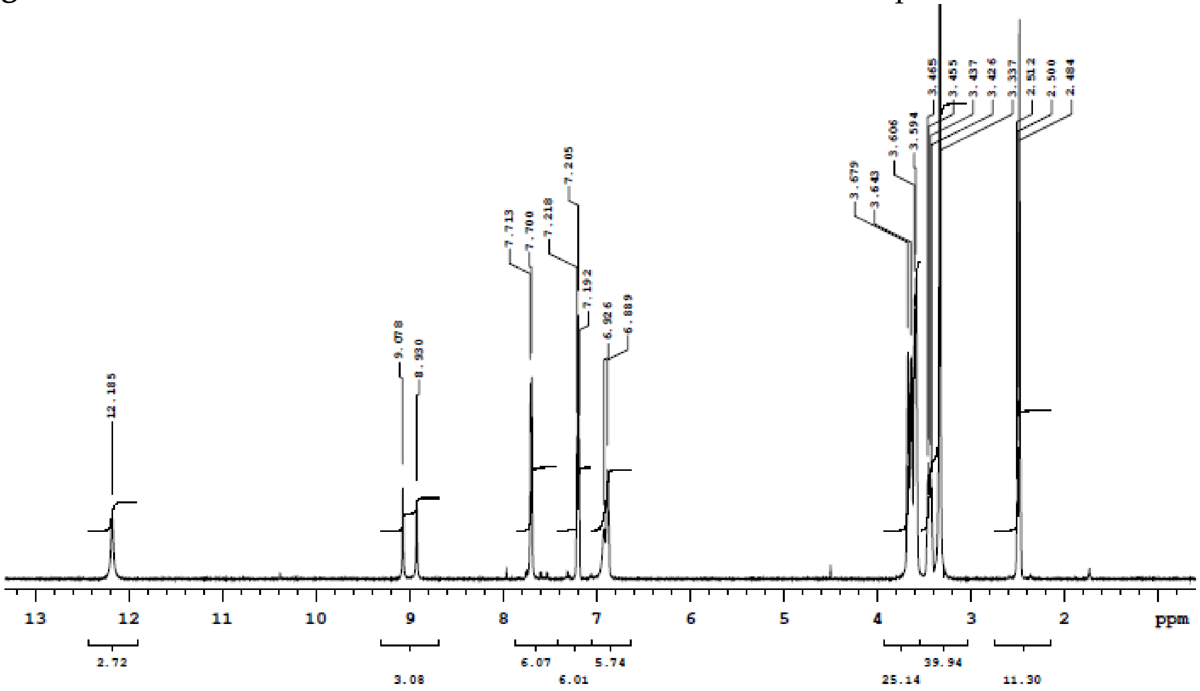

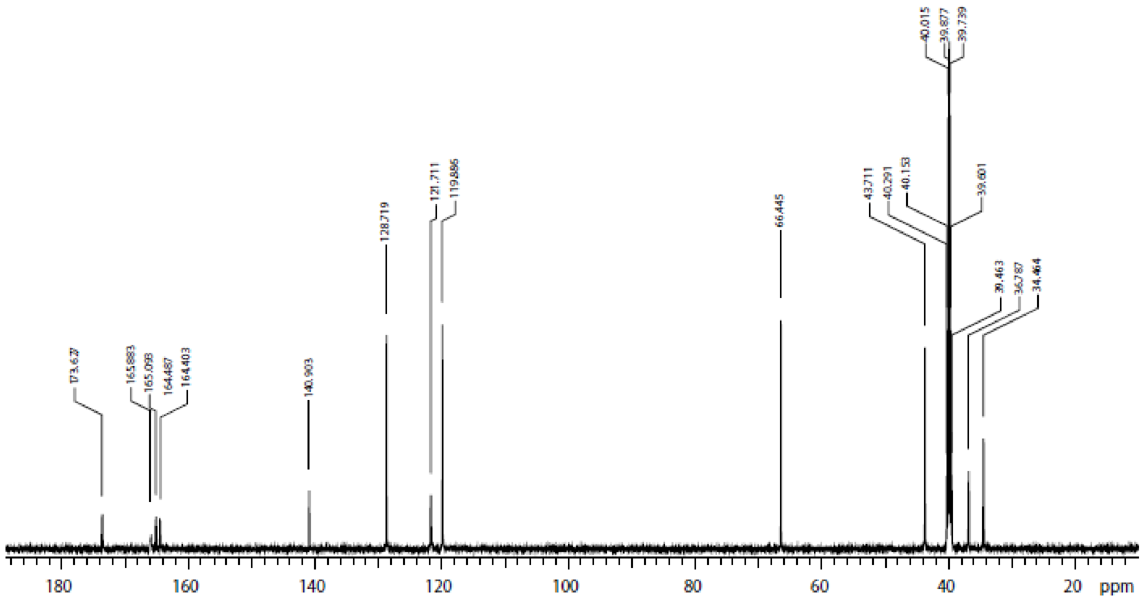

Figure S10 ^{1}H-NMR and ^{13}C-NMR for compound **5j**

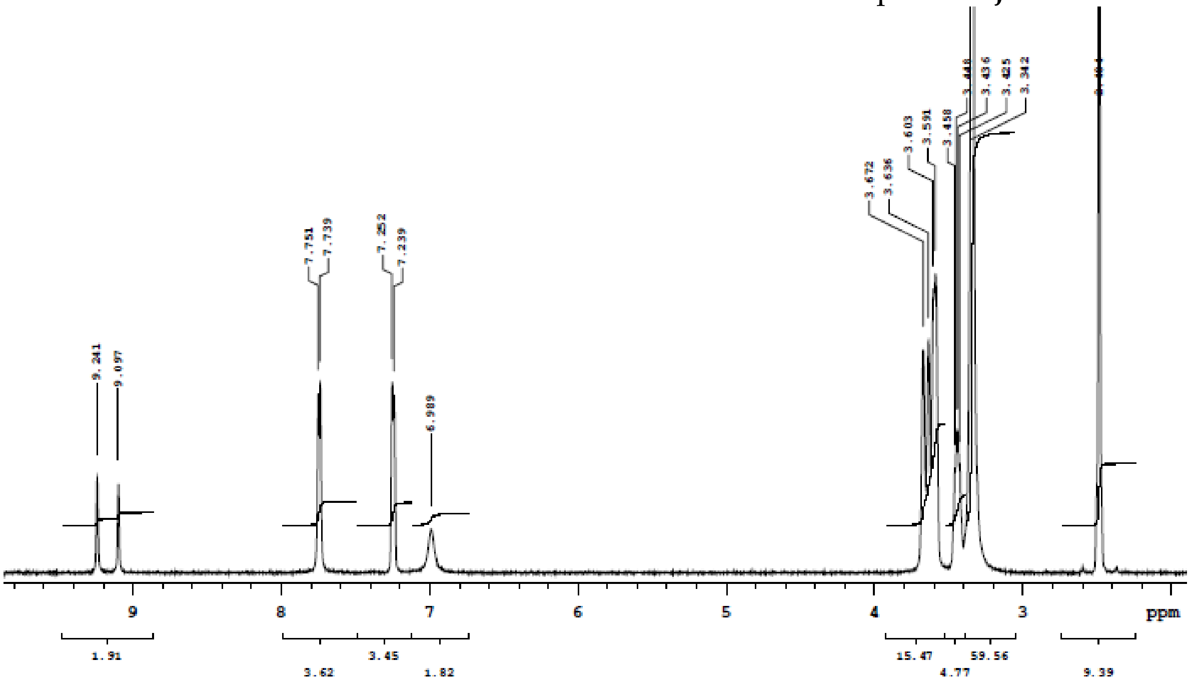

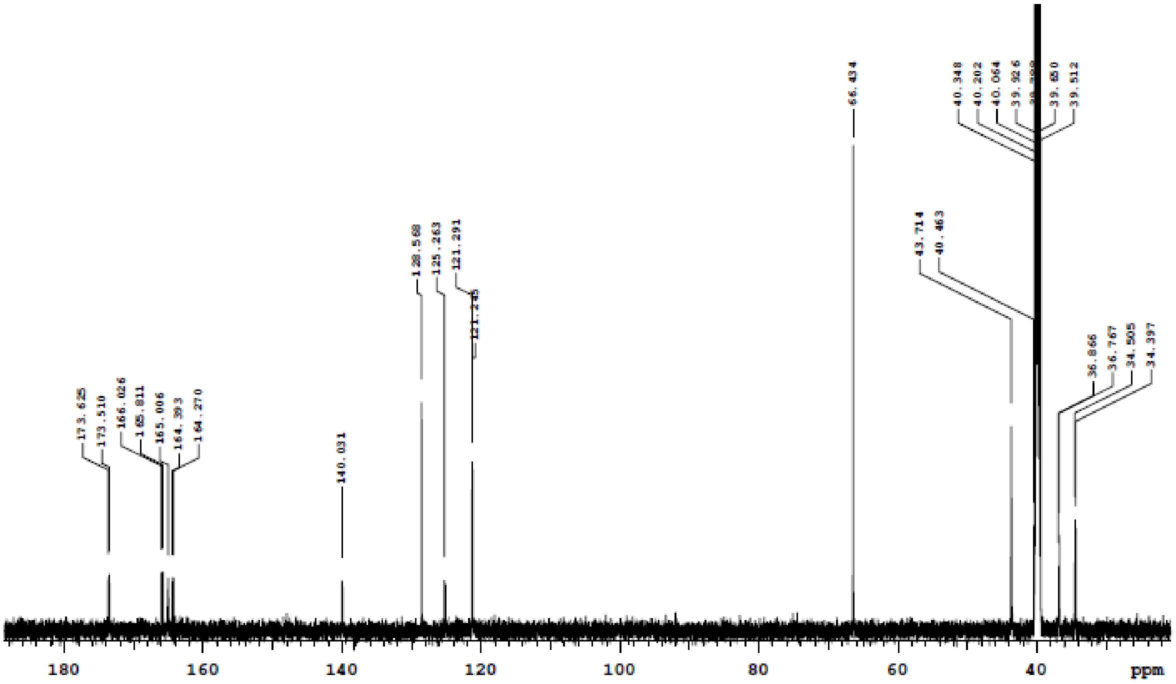

Figure S11 ^{1}H-NMR and ^{13}C-NMR for compound **5k**

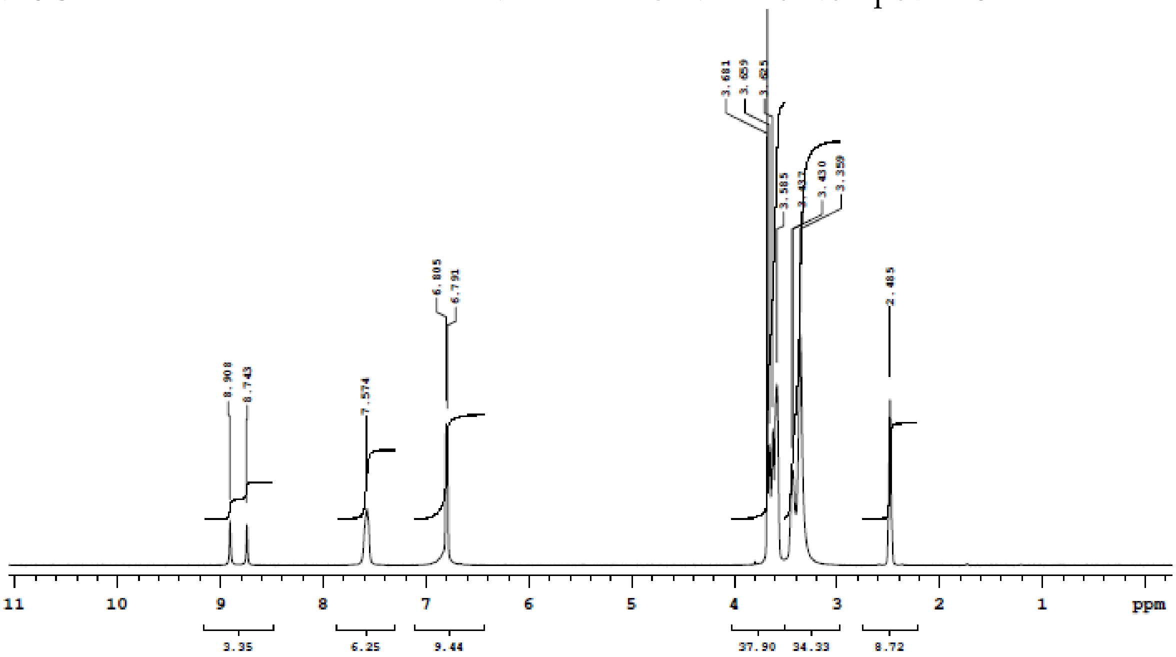

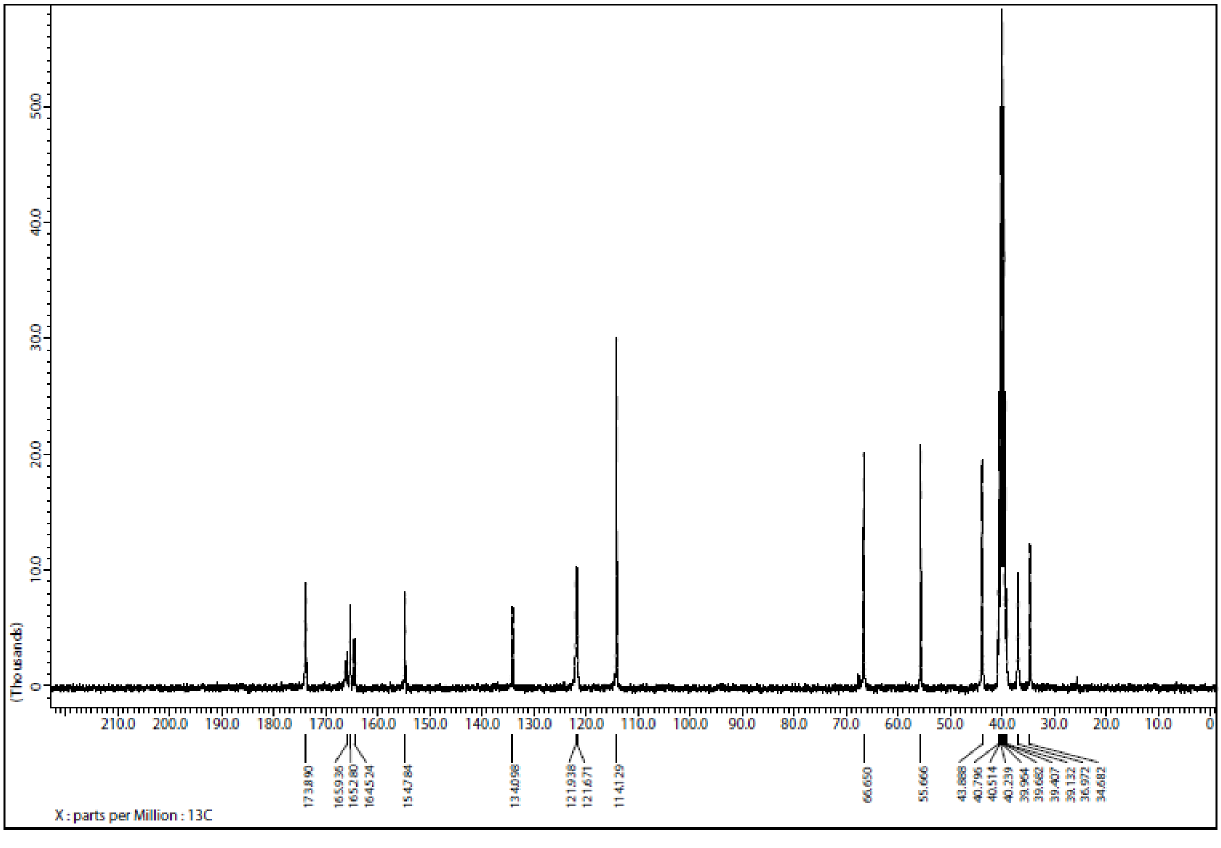

Figure S12 ^{1}H-NMR and ^{13}C-NMR for compound **5l**

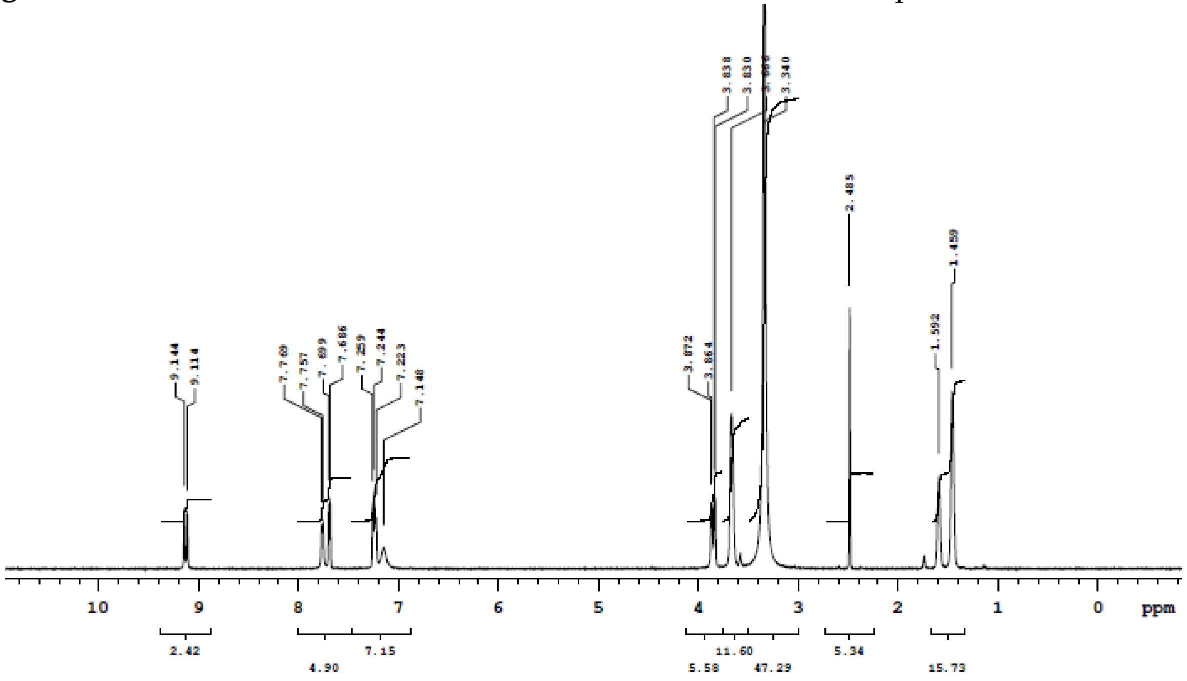

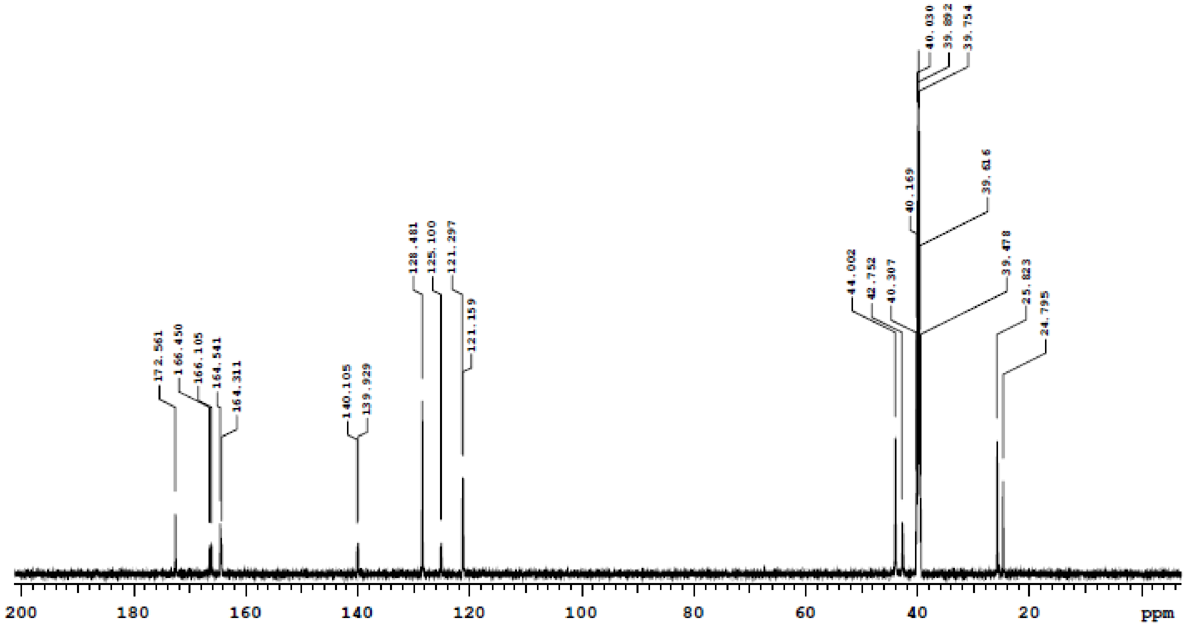

Figure S13 ^1H-NMR and ^{13}C-NMR for compound **5m**

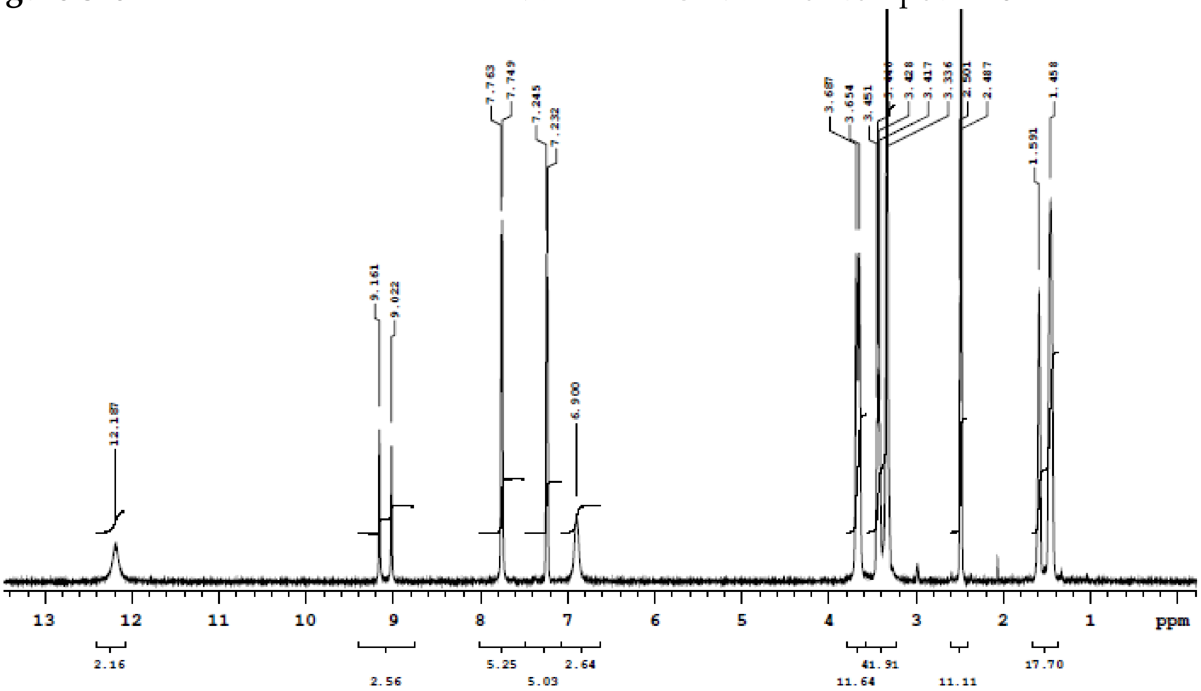

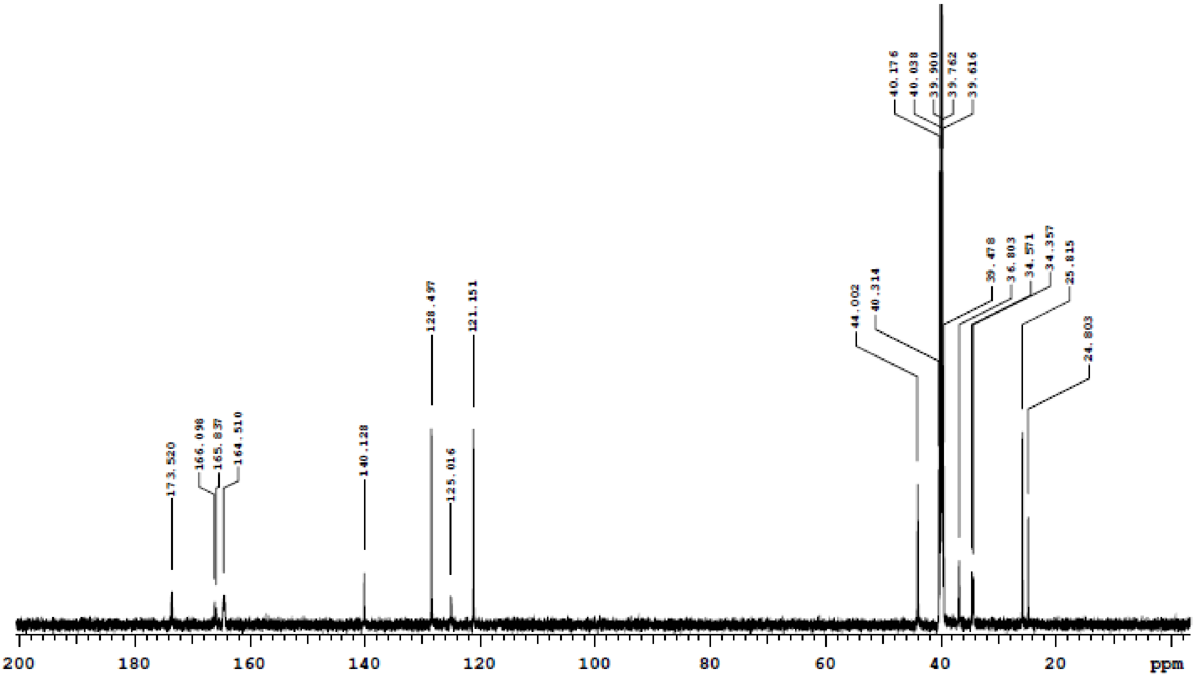

Figure S14 ^{1}H-NMR and ^{13}C-NMR for compound **5n**

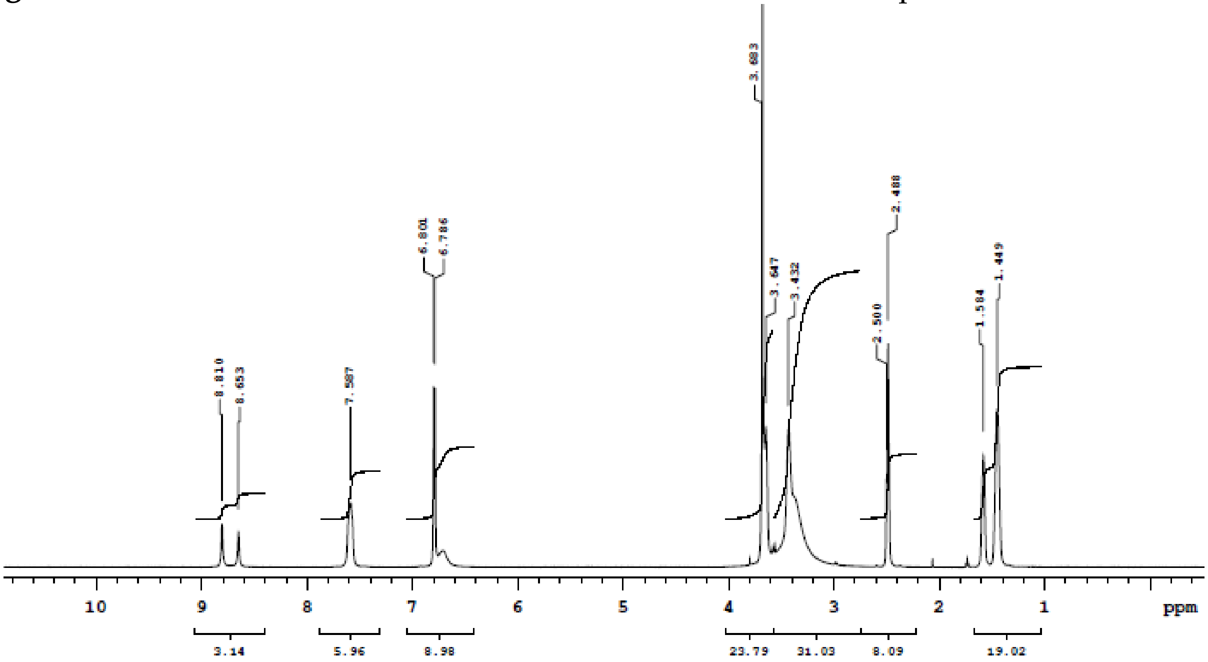

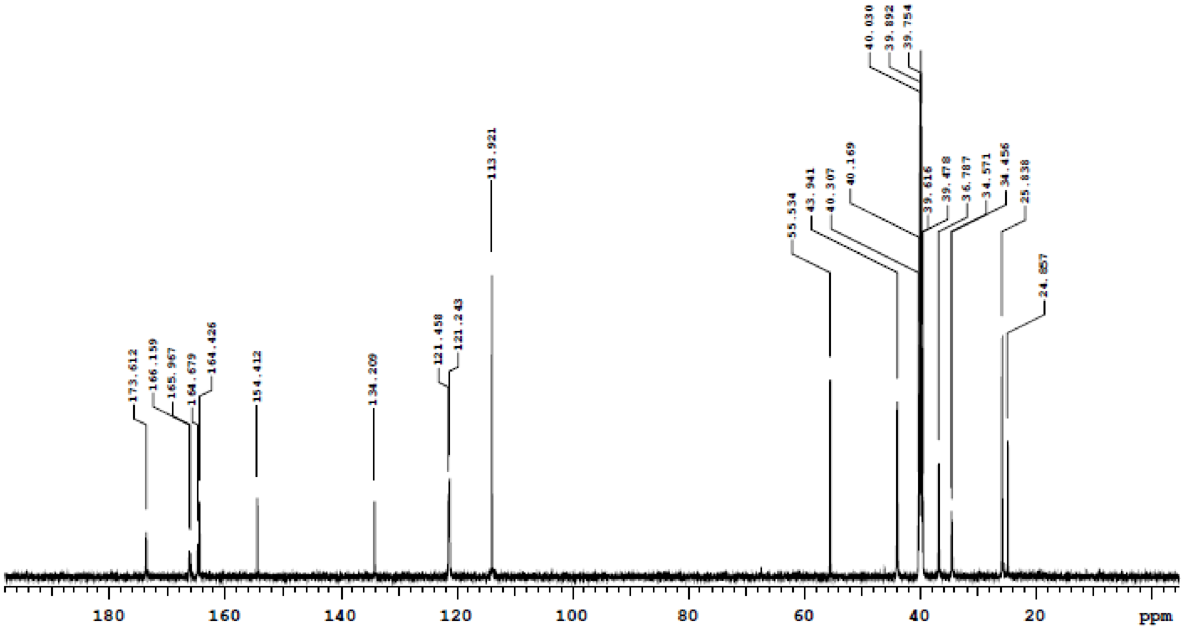

Figure S15 ^{1}H-NMR and ^{13}C-NMR for compound **5o**

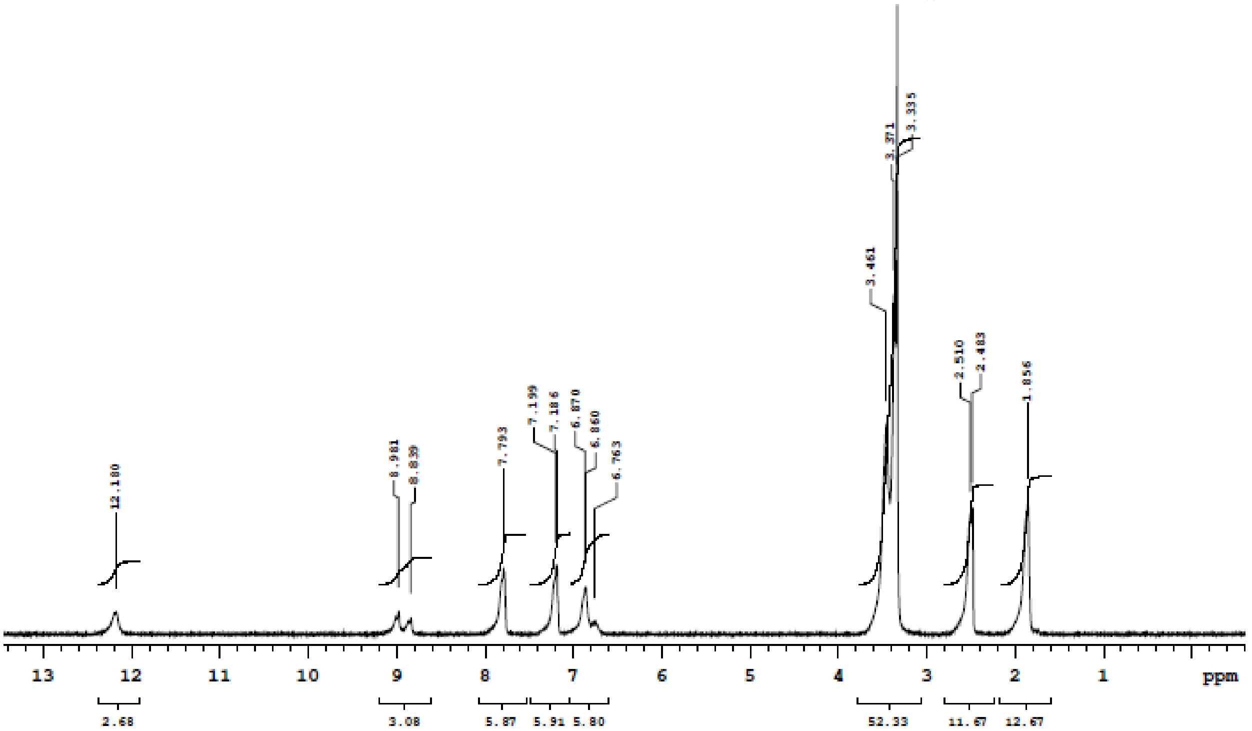

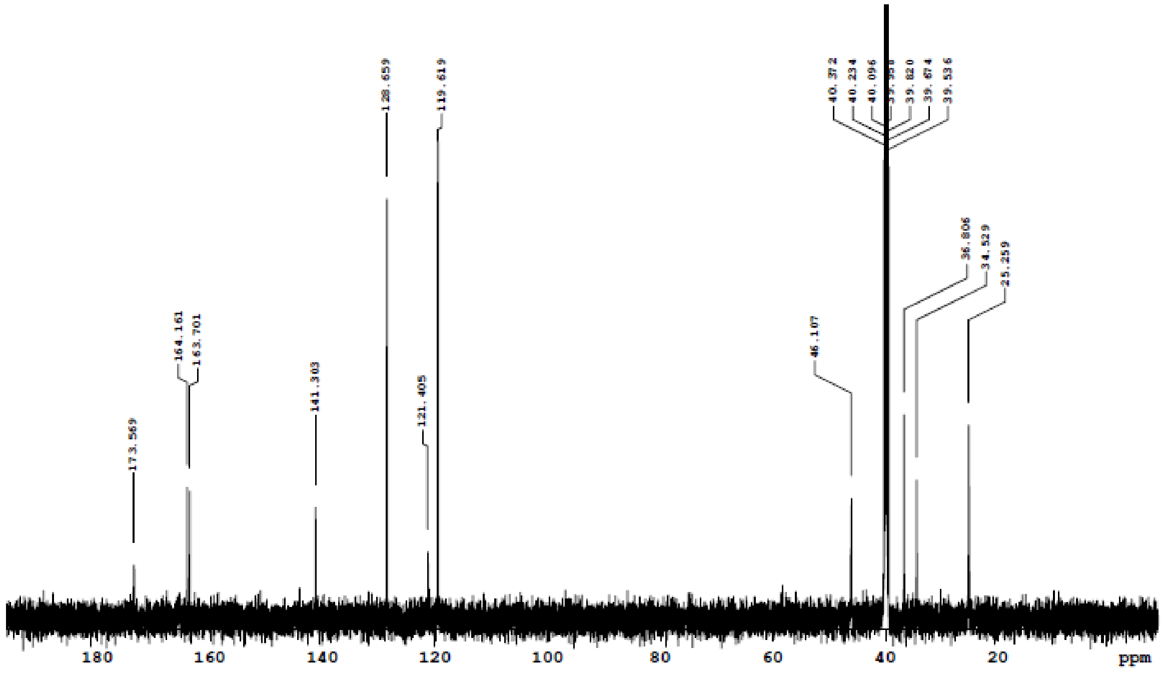

Figure S16 ^{1}H-NMR and ^{13}C-NMR for compound **5p**

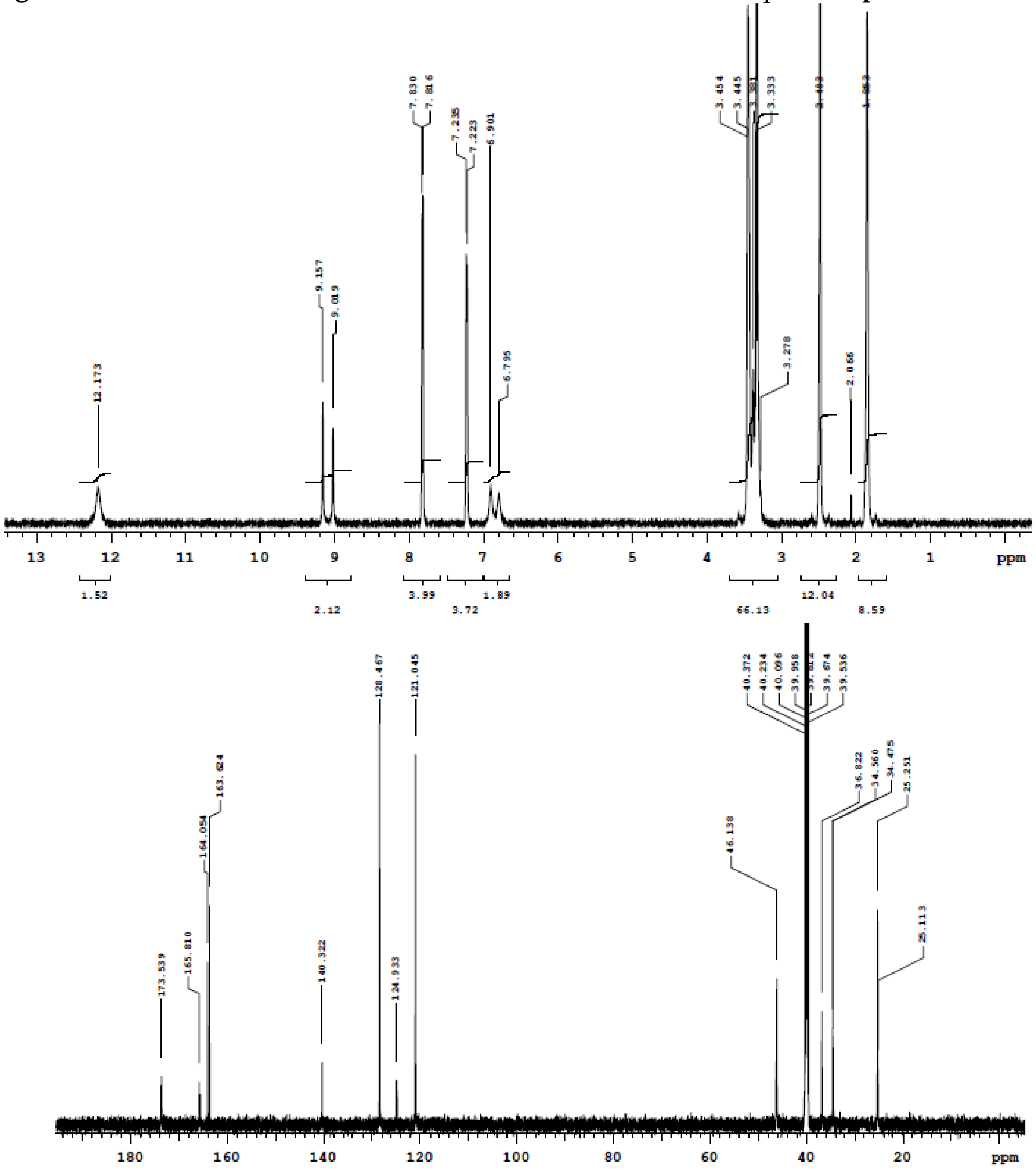

Figure S17 ^{1}H-NMR and ^{13}C-NMR for compound **5q**

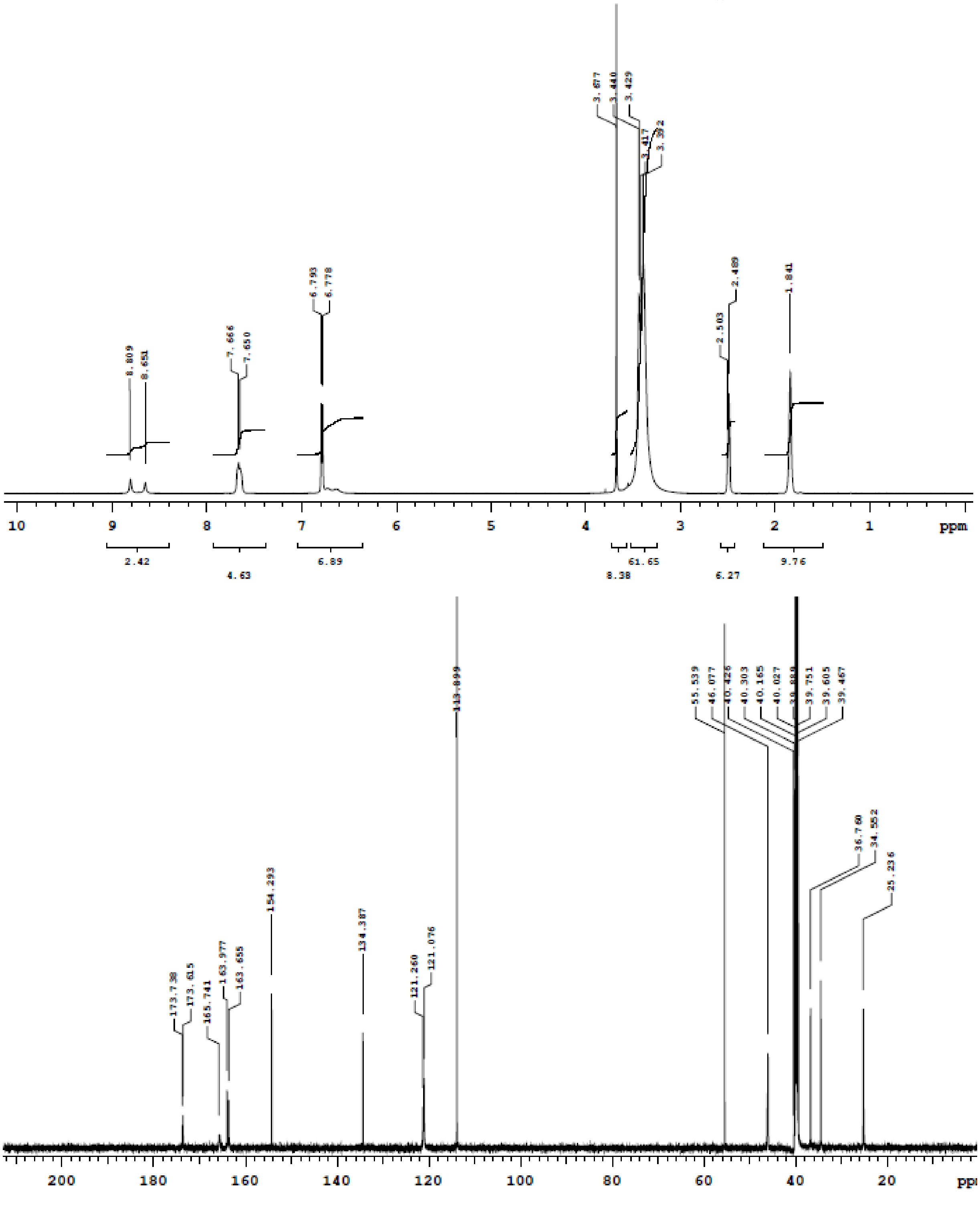

Supplement: Supplementary file 1 [file jof-06-00237-s001.pdf]
